# Development of the machine learning and deep learning models with SHAP strategy for predicting groundwater levels in South Korea

**DOI:** 10.1038/s41598-025-19545-y

**Published:** 2025-10-10

**Authors:** Sungwon Kim, Meysam Alizamir, Salim Heddam, Sun Woo Chang, Il-Moon Chung, Ozgur Kisi, Christoph Kulls

**Affiliations:** 1 Department of Railroad Construction and Safety Engineering, Dongyang University, Yeongju, 36040 Republic of Korea; 2 Institute of Research and Development, Duy Tan University, Da Nang, Vietnam; 3 School of Engineering and Technology, Duy Tan University, Da Nang, Vietnam; 4 Faculty of Science, Agronomy Department, Hydraulics Division, Laboratory of Research in Biodiversity Interaction Ecosystem and Biotechnology, University 20 Août 1955, Route El Hadaik, BP 26, Skikda, Algeria; 5 Department of Hydro Science and Engineering Research, Korea Institute of Civil Engineering and Building Technology, Goyang-si, Republic of Korea; 6 Department of Land, Water and Environmental Research, Korea Institute of Civil Engineering and Building Technology, Goyang-si, Republic of Korea; 7 Department of Civil Engineering, University of Applied Sciences, 23562 Lübeck, Germany; 8 Department of Civil Engineering, Ilia State University, 0179 Tbilisi, Georgia; 9 School of Civil, Environmental and Architectural Engineering, Korea University, Seoul, 02841 South Korea

**Keywords:** Groundwater levels, Machine learning, Deep learning, One-way ANOVA, SHAP, Environmental sciences, Hydrology

## Abstract

In this research, the groundwater levels (GWLs) were predicted by employing machine learning (i.e., stochastic gradient boosting (SGB), random forest (RF), generalized regression neural networks (GRNN), and group of method data handling (GMDH)) and deep learning (i.e., deep echo state network (Deep ESN) and long short-term memory (LSTM)) based on three predictive scenarios, Jeju Island, South Korea. In scenario 01, GWLs in Bongseong well was calculated utilizing rainfall, air temperature, relative humidity, wind speed, and various GWLs in different wells. Based on scenario 02, GWLs in Bongseong well was calculated using rainfall, air temperature, relative humidity, wind speed, and groundwater data (i.e., temperature, electric conductivity, and pressure). Finally, considering scenario 03, GWLs in Bongseong well were calculated by employing rainfall, air temperature, relative humidity, wind speed, and GWLs from 1-day to 15-day lead time. Five evaluation measures, including root mean squared error (RMSE), correlation coefficient (CC), Nash–Sutcliffe efficiency (NSE), relative error (RE), and root relative squared error (RRSE), were reflected for the predictive accuracy of developed models. Results showed that RF3 (RMSE = 0.053 m, CC = 1.000, NSE = 1.000, RE = 1.114, and RRSE = 0.013) based on scenario 03 performed the best predictive accuracy in GWLs of Bongseong well. Furthermore, the additional contributions of this research were achieved by the enhanced comparative evaluation through the SHapley Additive exPlanations (SHAP) strategy and one-way Analysis of Variance (ANOVA) test. The sensitivity analysis utilizing the SHAP strategy determined the significant feature indicator (i.e., GWL in 1-day lead-time) explaining its contribution to the predictive ability of developed models. The results of one-way ANOVA test provided that the predicted values were extracted from the same population as the measured values based on all models in scenario 03.

## Introduction

Groundwater remains essential for providing drinking water and supporting agricultural and industrial activities, especially in areas that lack sufficient surface water resources. The volcanic island (Jeju Island) in South Korea relies on groundwater as its main freshwater source because of its distinctive hydrogeological features. The combination of rising demands, unsustainable extraction practices, and climate variability has caused substantial changes in groundwater levels (GWLs) leading to future water security worries.

Groundwater below the earth’s surface corresponds to natural groundwater and is stored in various pores of soil and rock in aquifers within the geology. Groundwater is a source of river water recharge and is an essential element in the industrialization process and the supply of drinking water^1,2^. Monitoring has been conducted to check the overuse and depletion of groundwater, and groundwater quantity can be managed by observing GWLs. Many aspects of daily life depend heavily on groundwater below the surface where soil or rock is saturated, so careful management of groundwater is required. With continuous and technological advancement, machine learning (ML) and deep learning (DL) models have been utilized to manage groundwater and predict GWLs effectively^3,4^.

The GWLs, an indicator of available groundwater quantity within an aquifer, are affected by climatic elements and human actions^5–7^. Distillation of groundwater for diverse purposes, including industrial development, irrigation, freshwater supply, and urbanization, reduces groundwater reserves within the aquifer, thereby reducing GWLs^5^.

Overuse or mismanagement of groundwater to supply water for living, industrial, agricultural, and urban purposes can lead to a number of serious problems, including water shortages, deteriorating water quality, reduced crop yields, and land subsidence^8,9^. Additionally, the increase in the number of people dependent on groundwater and the high rate of industrialization cause excessive extraction and mismanagement of groundwater.

Groundwater systems are dynamic and respond to changes continuously in land use, groundwater extraction, and climate change. It is suggested that strategies for managing groundwater resources depend on several components, including utilization and convenience of correct data, financing, and policy implementation. However, another necessary component for operating groundwater resources is a precise estimation of GWLs. A monitoring system for reliable groundwater estimation is necessary for adequate groundwater preservation, such as arid and semi-arid regions vulnerable to drought^9,10^.

An accurate and reliable groundwater estimation system helps with short- and long-term preparation of sustainable groundwater distillation and repository. Additionally, accurate groundwater estimation helps to determine factors that affect optimization, such as groundwater recharge, discharge, storage, and infrastructure operation^11^. By optimally consuming and appropriately managing groundwater resources, environmental problems, including droughts, floods, famines, and landslides, can be mitigated or avoided^9^.

Over the past few decades, many GWL calculation and prediction techniques have been proposed that can be helpful in the operation of groundwater resources. However, the complex dynamics and heterogeneous nature of groundwater flow present challenges to accurate and understandable simulations. In addition, data uncertainty in various academic fields, including hydrology, climatology, hydrogeology, and meteorology etc., complicates the optimal process of numerical data^12^.

Most advanced level of scientific approaches for collecting long-term groundwater flow and remote data have helped to choose the optimal technique for analyzing and interpreting groundwater flow^13^. Also, with recent advancements in science and technology, many scientists, developers, and researchers are developing and applying diverse neuro-inspired models to reduce the uncertain restraints of conventional and historic models, such as physical, mathematical, and statistical-based models^14^.

The progress of this research is structured as follows. Chapter 2 explains various previous research on the calculation and prediction of GWLs. Chapter 3 presents diverse machine learning and deep learning models applied in this research. Chapter 4 provides the research area, data, and evaluation measures. Chapter 5 organizes the results of GWLs prediction utilizing the machine learning and deep learning models, SHapley Additive exPlanations (SHAP) strategy, and one-way Analysis of Variance (ANOVA) test. Chapter 6 discusses the importance, meaning, and relevance of machine learning and deep learning models. Finally, the main conclusions are addressed in Chap. 7.

## Reviewing previous researches for predicting GWLs

In the past, a lot of researches were conducted on calculating and predicting GWLs utilizing physical-based and universal models. Sahoo and Jha^15^ employed the multiple linear regression (MLR) model to predict GWLs in unconfined aquifer systems, Japan. Yousefi et al.^16^ utilized MODFLOW 2005-NWT model to predict long-term (10 years) GWLs, Iran. However, a physically-based model (i.e., MODFLOW 2005-NWT) is required to provide a large amount of data for predicting GWLs because of nonlinear relationships between different indicators in groundwater systems and other hydrological systems. Therefore, various neuro-inspired approaches, including machine learning and deep learning, have been employed by many researchers to overcome the limitations of physical models^17^.

Among neuro-inspired approaches, machine learning and hybrid machine learning models are increasingly important for predicting GWLs because of their ability to make reliable and accurate predictions, learn from prior calculations, and adapt to new data^2^. Huang and Tian^18^ developed artificial neural networks (ANN), support vector machines (SVM), and M5Tree models to predict GWLs, China. Results showed that M5Tree (Huanghuaying station) and SVR (Shuguang station) models provided the best performance for predicting GWLs on different stations. Sattari et al.^19^ utilized SVR and M5Tree models to predict the changes in GWLs, Iran. They indicated that SVR model was superior to M5Tree model for predicting the changes in GWLs. Takafuji et al.^13^ employed sequential Gaussian simulation (SGS) to predict GWLs in 49 monitoring wells, Brazil, and compared the results with ANN and autoregressive integrated moving average (ARIMA) models. In addition, Osman et al.^20^ applied various ensemble techniques to predict GWLs, Malaysia. Among them, the extreme gradient boosting (XGBoost) model was determined to be the most powerful model. Kouziokas et al.^21^ implemented various ANN models, combining resilient backpropagation (RB-ANN), Levenberg Marquardt (LM-ANN), scaled conjugate gradient (SCG-ANN), and BFGS Quasi-Newton (BFGSQN-ANN), to predict GWLs, USA. Results explained that LM-ANN model was found to be the most reliable model for predicting GWLs. Li et al.^5^ combined the different optimization approaches and ANN, such as artificial bee colony (ABC-ANN), particle swarm optimization (PSO-ANN), genetic algorithm (GA-ANN), and standalone ANN models, to predict GWLs, China. As a result, the ABC-ANN model provided the best results for predicting GWLs. Banadkooki et al.^22^ employed radial basis function neural networks embedded whale algorithm (WA-RBFNN), ANN embedded whale algorithm (WA-ANN), and genetic programming (GP) models to predict GWLs, Iran. Among the employed models, WA-ANN model showed the best results for predicting GWLs. Yadav et al.^23^ estimated GWLs utilizing singular spectrum analysis (SSA), mutual information (MI), GA, ANN, and SVM models, India. Results demonstrated that SSA-MI-GA-ANN and SSA-MI-GA-SVM models derived more accurate results for predicting GWLs than ANN and SVM models. Choubin and Rahmati^24^ administered simulated annealing (SA) to random forest (RF) model, and predicted GWLs to a high degree of accuracy. Yadav et al.^10^ measured monthly GWLs based on extreme learning machine (ELM), DL, and SVM models at two locations, Canada. Among the indicators available, prior GWL data was found to have the greatest influence on the model’s performance. Results explained that ELM model was found to be the best.

Moreover, rapid advancements in neuro-inspired approaches have enabled the application of deep learning and hybrid deep learning models to accurately predict GWLs. Feng et al.^25^ investigated diverse deep learning models for predicting GWLs, Iran. They found that convolutional neural networks (CNN) supplied the superior performance compared to rival deep learning (i.e., generative adversarial networks (GAN) and recurrent neural networks (RNN)) and machine learning (i.e., SVM, RF, and decision tree (DT)) models. Mirboluki et al.^26^ integrated grey wolf optimization (GWO) and long short-term memory (LSTM) for developing hybrid model to predict GWLs, Iran. The predictive accuracy of developed hybrid model (i.e., LSTM-GWO) was compared to ANN-GWO and standalone ANN. Results showed that LSTM-ANN was superior to standalone ANN and ANN for predicting GWLs.

Chang et al.^27^ combined CNN with backpropagation (BP) for predicting GWLs in 25 monitoring stations, Taiwan. Results showed that CNN-BP outperformed BP for predictive accuracy of GWLs. Tiwari^28^ employed machine learning (i.e., RF and SVM), deep learning (i.e., LSTM and bidirectional LSTM (BiLSTM)), and hybrid deep learning (RF-LSTM) models for predicting GWLs. As a result, RF, a machine learning model, performed the best accuracy for predicting GWLs compared to above-mentioned models.

In machine learning and deep learning models, the procedure of choosing specific model for accurate performance from the group of potential models is defined as model selection^29^. In this research, among various neuro-inspired approaches, four machine learning and two deep learning models, which were not relatively frequent utilization for predicting GWLs issues in previous literatures, were selected for predicting GWLs in Bongseong well, Jeju Island, South Korea. That is, this research aims to demonstrate the superiority of machine learning and deep learning models that are not commonly utilized for predicting GWLs issues.

Also, three scenarios were applied to configure various input layers. The dataset utilized were time series data on GWLs collected from neighbor wells, including the Bongseong well, data on various groundwater indicators, and meteorological data from Aewol (1) and Witse Oreum stations. In addition, the predictive performance of developed models was evaluated by comparing five evaluation measures and diverse visual assistances. Also, predictive results of this research demonstrated by the augmented comparative evaluation including the SHAP strategy and one-way ANOVA test.

## Neuro-inspiring approaches

### Machine learning model

Machine learning model (MLM) is an area of research and study in neuroscience regarding the implementation of statistical description that can train from the dataset and generalize to the unnoticed dataset and accomplish projects without accurate information^30^. Recently, MLM has outperformed many physical and mathematical models in prediction and forecasting problems^31–33^. MLM has been employed in many physical science and engineering fields, including hydrology, hydraulics, water resources, water quality, groundwater, agriculture, soil temperature and so on^4,34^.

**Fig. 1:**
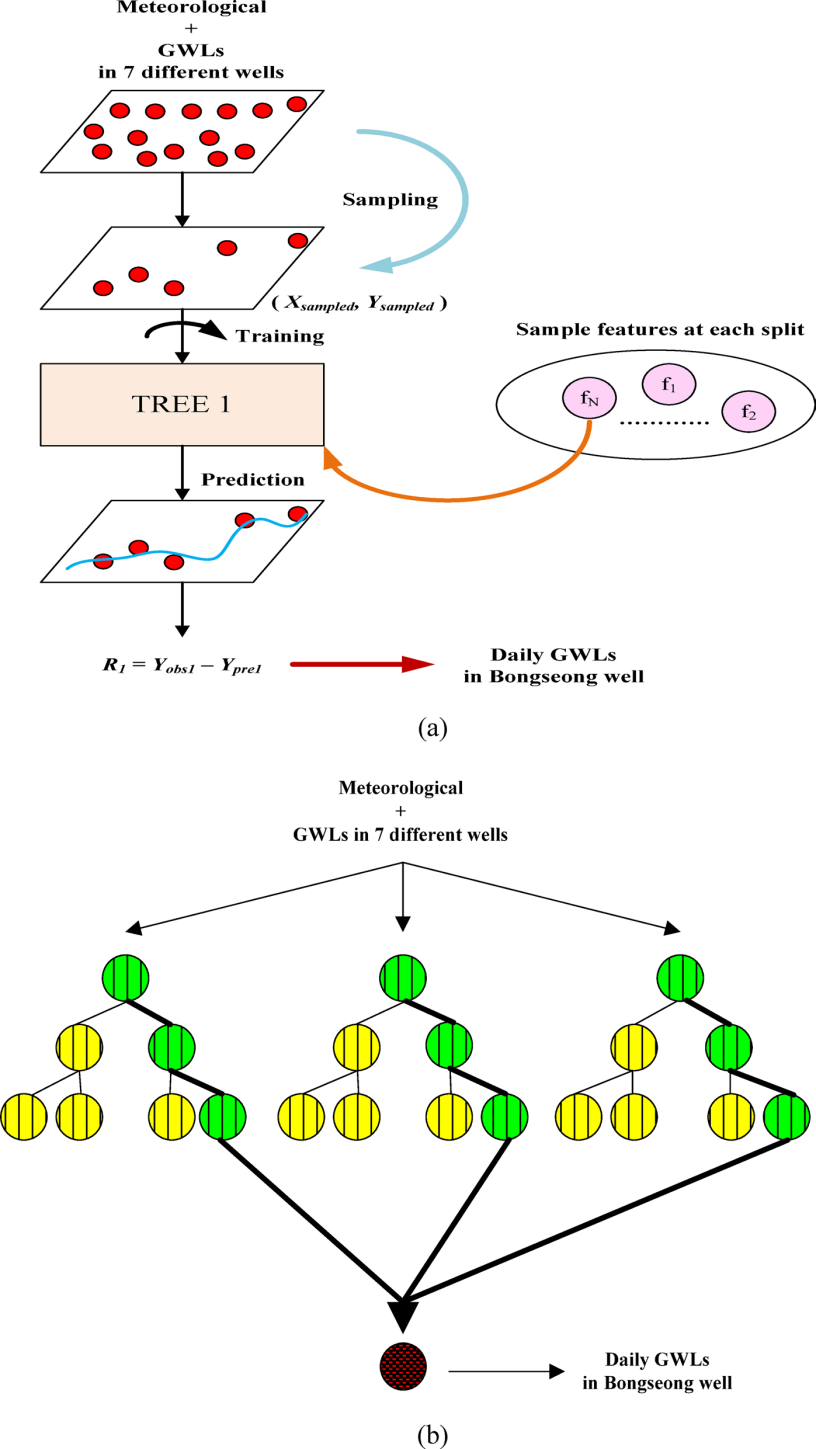

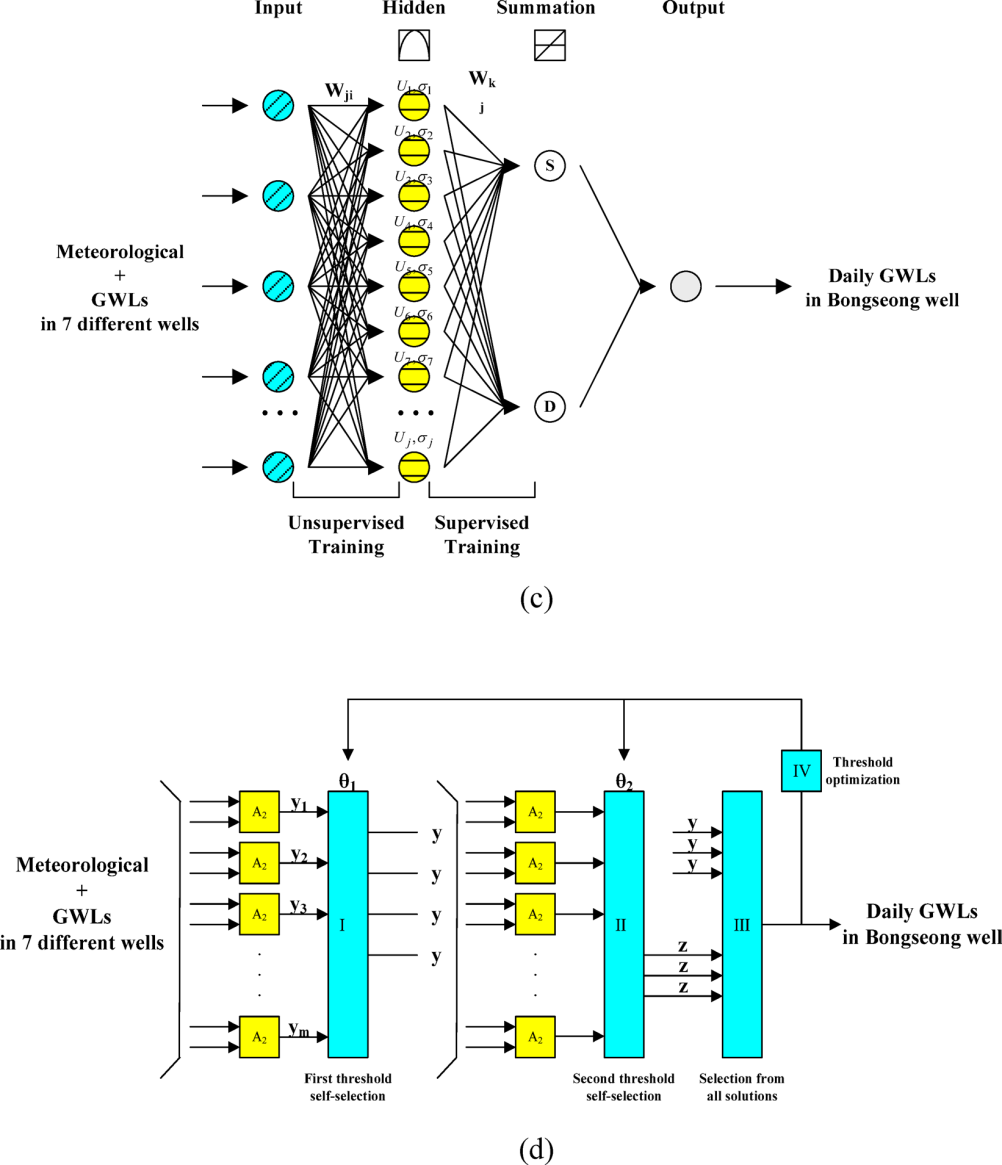
Architecture of machine learning models based on scenario 01. (**a**) SGB, (**b**) RF, (**c**) RF, (**d**) GMDH.

In the addressed research, four machine learning models were employed: stochastic gradient boosting (SGB), random forest (RF), generalized regression neural networks (GRNN), and group method of data handling (GMDH). The four employed machine learning models have been frequently implemented in similar and diverse research fields. They have been applied to solve various linear and nonlinear calculation and prediction problems in many fields and have provided accurate performance of machine learning models. Therefore, detailed descriptions of SGB^33,35–37^, RF^38–41^, GRNN^30,42–45^, and GMDH^46–49^ models are replaced with the suggested references of MLMs. Also, the architecture of each MLM for scenario 01 applied in the addressed research was presented in the following visual representation including Figs. 1(a)-(d).

### Deep learning model

The deep learning model (DLM) is the sub-group of MLMs based on neuroscience with depiction training. Deep means implementing multiple hidden layers in the embedded networks^50^. DLM can be trained based on unsupervised and supervised algorithms^51^. The architectures of various DLMs, such as deep echo state network (Deep ESN), deep neural networks (Deep NN), deep belief networks (Deep BN), long short-term memory (LSTM), recurrent neural networks (RNN), and convolutional neural networks (CNN), have been employed to fields including geoscience, bioinformatics, medical image classification, climatic science, where they have provided outputs exceeding performance of human experts in some case study and so on^33,50,52,53^.

**Fig. 2:**
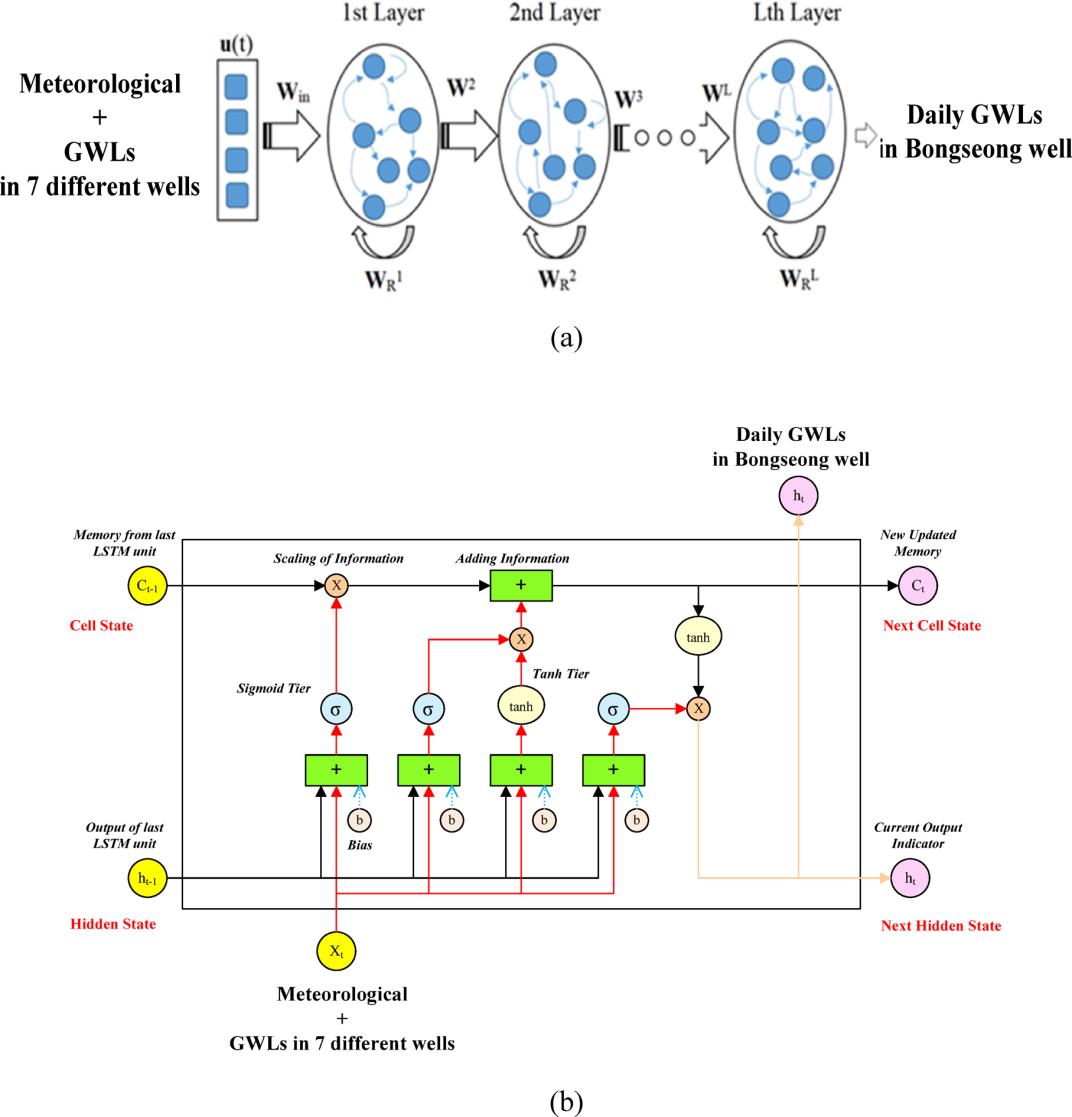
Architecture of deep learning models based on scenario 01. (**a**) Deep ESN, (**b**) LSTM.

In the addressed research, GWLs in Bongseong well were predicted to employ Deep ESN and LSTM models, which have been widely utilized in the previous research of hydrology and water resources fields. Detailed description and explanation of Deep ESN^2,52,54–56^ and LSTM^57–60^ models are neglected and replaced with provided references. In addition, the architecture of Deep ESN and LSTM models based on scenario 01 employed in the addressed research was provided in the following optical images including Figs. 2(a)-(b).

## Case study

### Selection of monitoring wells and data

**Fig. 3:**
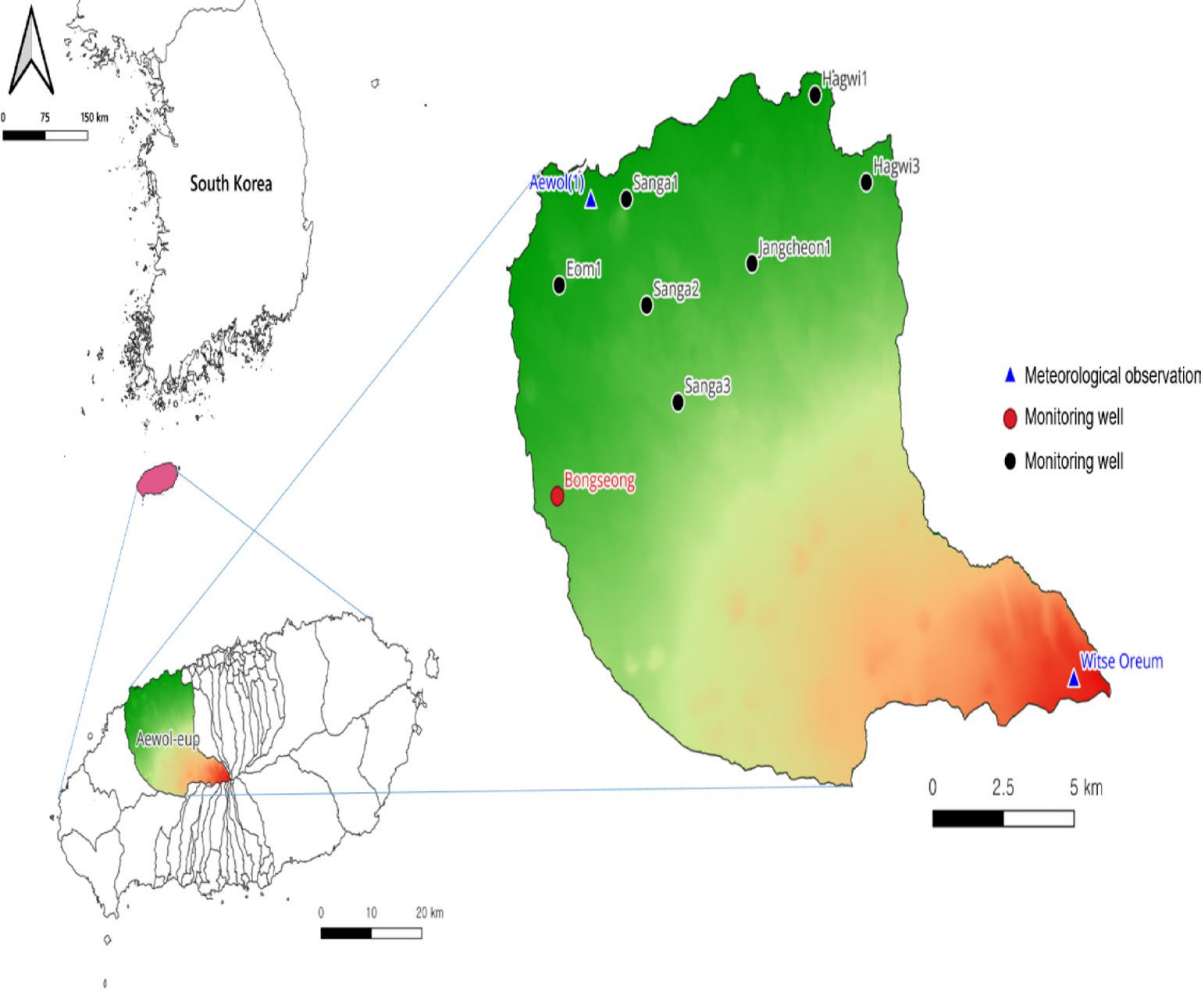
Schematic map of selected monitoring wells and meteorological stations. Schematic map of selected monitoring wells and meteorological stations created by the authors utilizing the QGIS program (version 3.34), which is open-source, freely available geographic information system (GIS) software (https://qgis.org).

In the addressed research, eight monitoring wells were selected as the research groundwater wells for calculating and predicting GWLs in Aewol-eup, Jeju Island. Eight monitoring wells selected are composed of Bongseong, Sanga1, Sanga2, Sanga3, Eom1, Jangcheon1, Hagwi1, and Hagwi3 wells at the Aewol-eup. Table 1 explains the geological characteristics of selected monitoring wells in this research. We can find from Table 1 that the depth of the Sanga3 well is 350 m, which is the deepest, and the depth of the Hagwi1 well is 120 m, which is the shallowest among the monitoring wells. Also, the casing diameter of eight monitoring wells was found to be the same, 200 mm. In addition, topographic data for various meteorological stations around Aewol-eup utilized can be provided in Table 2. Daily rainfall data was collected from the Aewol (1) station installed at the Aewol-eup office. Also, meteorological data on daily air temperature, relative humidity, and wind speed were assembled from the Witse Oreum station at the Witse Oreum shelter. Figure 3 provides the schematic map of selected monitoring wells and meteorological stations in the addressed research. It was created by the authors utilizing the QGIS program (version 3.34), which is open-source, freely available geographic information system (GIS) software (https://qgis.org). The boundary data implemented in Fig. 3 employed open spatial information data provided by the Public Data Portal (https://www.data.go.kr). It is distributed under an open license that permits free use, including modification and publication. Therefore, no separate copyright permission is required.

**Table 1:** Geological characteristics of selected monitoring wells in this research.

Well	Location	Elevation (m)	Longitude	Latitude	Excavation Depth (m)	Casing Diameter (mm)
Bongseong	Aewol	167.2	126°19’06.81”	33°23’58.10”	200.0	200
Sanga1	Aewol	50.0	126°20’34.55”	33°27’43.86”	150.0	200
Sanga2	Aewol	145.5	126°21’04.73”	33°26’24.55”	175.0	200
Sanga3	Aewol	250.0	126°21’49.86”	33°25’12.37”	350.0	200
Eom1	Aewol	60.0	126°19’04.60”	33°26’37.09”	160.0	200
Jangcheon1	Aewol	131.1	126°23’28.04”	33°26’59.00”	160.0	200
Hagwi1	Aewol	13.0	126°24’50.64”	33°29’07.76”	120.0	100
Hagwi3	Aewol	116.0	126°26’02.70”	33°28’03.44”	140.0	100

**Table 2:** Geological characteristics of selected meteorological indicators.

Indicators	Unit	Elevation (m)	Longitude	Latitude	Station
Rainfall	mm	36.0	126°19’45.92”	33°27’43.28”	Aewol (1)
Air temperature	℃	1,673.0	126°30’57.56”	33°21’56.25”	Witse Oreum
Relative humidity	%	1,673.0	126°30’57.56”	33°21’56.25”	Witse Oreum
Wind speed	m/sec	1,673.0	126°30’57.56”	33°21’56.25”	Witse Oreum

The meteorological (i.e., rainfall, air temperature, relative humidity, and wind speed) and groundwater (i.e., temperature, conductivity, pressure, and GWLs) data utilized were selected from June 1, 2011 to December 31, 2020 on daily time scale, and the total number of data is 3,502. Among them, the data employed for the training procedure of MLMs and DLMs is 2,802, 80% of the total data from June 1, 2011 to January 31, 2019. Also, the data employed for the testing procedure of MLMs and DLMs is 700, which is 20% of the total data from February 1, 2019 to December 31, 2020. The collected data, including meteorological and groundwater parameters, for predicting GWLs in Bongseong well can be directly accessed and downloaded from the official website of Groundwater Information Management System, Jeju island (https://water.jeju.go.kr/obsvsystem/gwobsv/obsvData). The data presented in this research are available upon request from the corresponding author (contact ozgur.kisi@th-luebeck.de).

Table 3 indicates the training and testing data of meteorological (i.e., rainfall, air temperature, relative humidity, and wind speed) and groundwater (i.e., temperature, conductivity, pressure, and GWLs) in Bongseong well. They explain the average (AVE.), maximum (MAX.), minimum (MIN.), standard deviation (S_x_), coefficient of variation (C_v_), skewness coefficient (C_sx_), and standard error (SE) during training and testing procedure. As seen in Table 3, the value of standard deviation displays the highest rate for relative humidity and the lowest rate for groundwater temperature. The values for the coefficient of variation provide the highest rainfall rate, and the lowest rate in groundwater temperature and pressure. The values of the skewness coefficient display the largest rate in the rainfall data. In addition, the standard error demonstrates the maximum value in relative humidity and the minimum value in groundwater temperature. Also, the values of the skewness coefficient give negative values ​​in air temperature, relative humidity, and pressure in the training data. Testing data gives negative rates ​​in air temperature, relative humidity, groundwater temperature, and pressure.

**Table 3:** Basic statistical analysis of training and testing data utilized.

Division	Data	Indicator	Number	Unit	AVE.	MIN.	MAX.	S_x_	C_v_	C_sx_	SE
Training	Meteorological Data	Rainfall	2802	mm	3.23	0.00	278.50	12.11	3.75	10.16	0.23
		Air Temperature	2802	℃	6.42	-16.39	22.35	8.93	1.39	-0.37	0.17
		Relative Humidity	2802	%	75.00	5.79	100.00	22.91	0.31	-0.82	0.43
		Wind Speed	2802	m/sec	12.96	0.00	48.17	6.46	0.50	1.21	0.12
	Groundwater Data (Bongseong)	Groundwater Temperature	2802	℃	15.58	15.22	15.95	0.18	0.01	0.14	0.00
		Conductivity	2802	µS/㎝	173.49	140.21	233.24	20.24	0.12	0.23	0.38
		Pressure	2802	hpa	1,009.68	925.00	1,035.86	11.75	0.01	-1.06	0.22
		GWL	2802	m	9.81	4.88	17.52	2.79	0.28	0.49	0.05
Testing	Meteorological Data	rainfall	700	mm	4.10	0.00	176.50	14.41	3.52	6.05	0.54
		Air Temperature	700	℃	7.19	-12.71	21.78	7.79	1.08	-0.29	0.29
		Relative Humidity	700	%	67.53	5.96	99.96	23.44	0.35	-0.60	0.89
		Wind Speed	700	m/sec	13.28	3.89	43.27	6.83	0.51	1.36	0.26
	Groundwater Data (Bongseong)	Groundwater Temperature	2802	℃	15.65	15.23	15.86	0.13	0.01	-0.42	0.00
		Conductivity	700	µS/㎝	186.78	157.00	236.00	20.92	0.11	0.65	0.79
		Pressure	700	hpa	1,016.65	992.71	1,033.88	7.76	0.01	-0.11	0.29
		GWL	700	m	12.75	6.45	18.20	4.08	0.32	0.00	0.15

Ave. = average; Min. = minimum; Max. = maximum; S_x_ = standard deviation; C_v_ = coefficient of variation; C_sx_ = skewness coefficient; SE = standard error.

Table 4 describes the hyperparameters tuning for machine learning and deep learning models. In this research, the hyperparameters, which were utilized to the three scenarios (i.e., 01, 02, and 03), were applied for evaluating individual model. Therefore, the different selection of feature indicators was set to influence the predictive accuracy.

**Table 4:** Hyperparameters tuning for MLMs and DLMs.

Division	Model	Hyperparameters
Machine learning	SGB	max. of trees = 400, depth of individual trees = 5, min size node = 10, k-fold cross validation = 10
	RF	max. of trees = 200, max. tree levels = 50, min. size node = 2, tree validation method = out of bag (OOB)
	GRNN	min. σ = 0.0001, max. σ = 10, Kernel type = Gaussian, k-fold cross validation = 10
	GMDH	max. networks layers = 20, max. polynomial = 16, k-fold cross validation = 10
Deep learning	Deep ESN	neuron number for a reservoir = 300, a spectral radius = 0.95, input scaling = 0.5, leaking rate = 0.3, regularization parameter = 0.001
	LSTM	neuron number for single LSTM = 200, dropout rate = 0.01, recurrent dropout rate = 0.085, Adam optimizer = 300 epochs

**Fig. 4:**
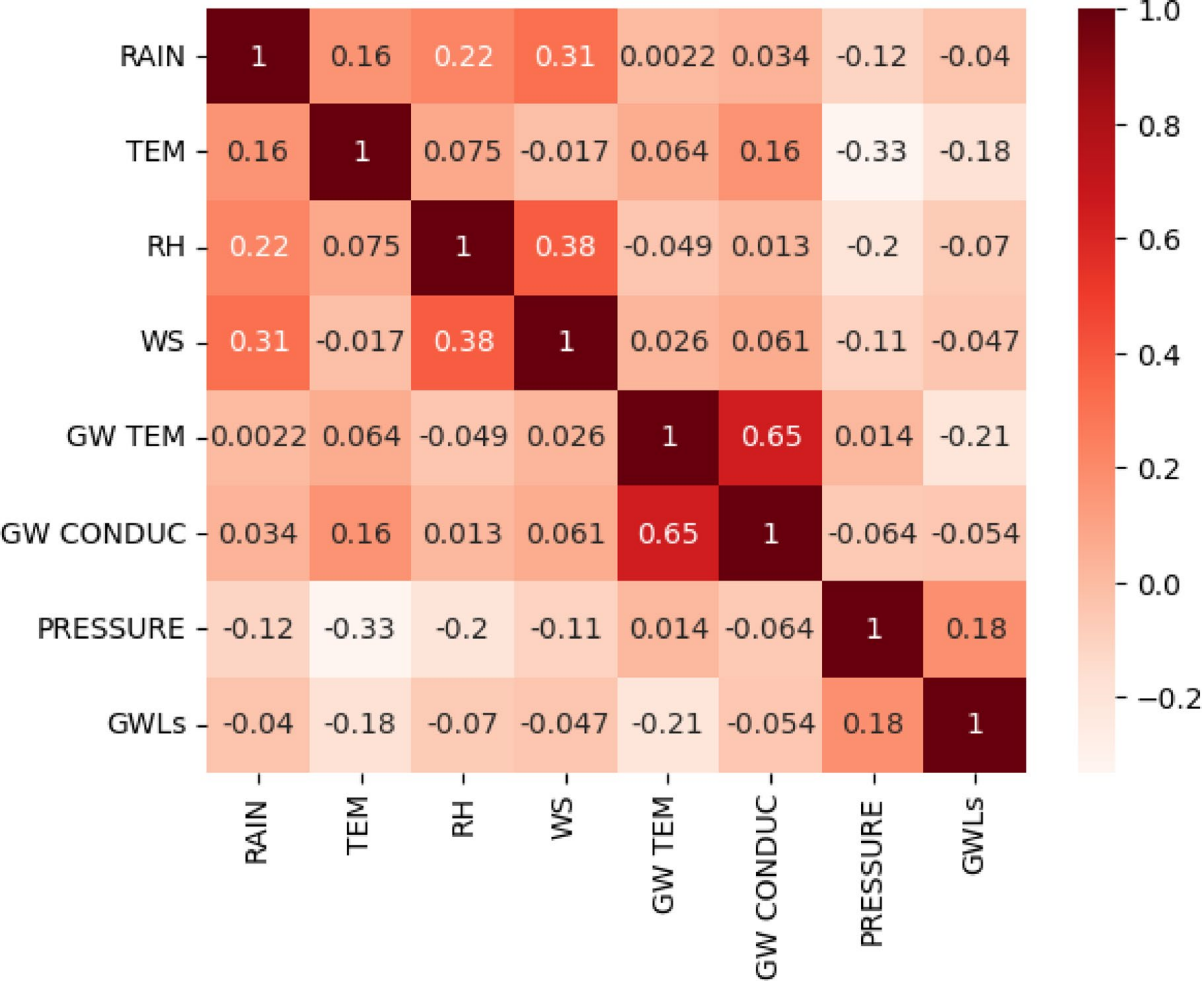
Heat map for the correlation between the feature (meteorological and groundwater data) and output (GWLs in Bongseong well).

Figure 4 displays a heat map for the correlation between independent indicators (i.e., meteorological and groundwater data) and GWLs in the Bongseong well. A heat map explains a two-dimensional visual approach that illustrates the magnitude of independent indicators with various colors. It can be found from Fig. 4 that GWLs in Bongseong well had the highest correlation coefficient with pressure of 0.18, and were analyzed to have negative values ​​with all other independent indicators except for pressure. Also, when resolving the correlation coefficient between various independent indicators, the correlation coefficient between groundwater conductivity and groundwater temperature was the highest at 0.65. Therefore, the dependence between the independent indicators was evaluated as a low condition in the addressed research.

### Evaluation measures for model performance

Evaluation of calculated and predictive results for the accomplishment of MLMs and DLMs can be effectively performed using specific evaluation measures based on training and testing procedures. The evaluation measures considered in the addressed research are root mean square error (RMSE), correlation coefficient (CC), Nash–Sutcliffe efficiency (NSE), relative error (RE), and root relative squared error (RRSE). These evaluation measures provide information about how well the model’s calculation and prediction capabilities fit the measured data, and can be expressed as Eqs. (1)-(5).

1 $$\:\text{R}\text{M}\text{S}\text{E}=\sqrt{\frac{{\sum\:}_{\text{i}=1}^{\text{n}}{\left(\right(\text{G}\text{W}\text{L}{)}_{\text{i}\text{o}}-\left(\text{G}\text{W}\text{L}{)}_{\text{i}\text{p}}\right)}^{2}}{\text{n}}}$$

2 $$\:\text{C}\text{C}=\frac{{\sum\:}_{\text{i}=1}^{\text{n}}({\left(\text{G}\text{W}\text{L}\right)}_{\text{i}\text{o}}-{\left(\stackrel{-}{\text{G}\text{W}\text{L}}\right)}_{\text{i}\text{o}})({\left(\text{G}\text{W}\text{L}\right)}_{\text{i}\text{p}}-{\left(\stackrel{-}{\text{G}\text{W}\text{L}}\right)}_{\text{i}\text{p}})}{\sqrt{{\sum\:}_{\text{i}=1}^{\text{n}}{({\left(\text{G}\text{W}\text{L}\right)}_{\text{i}\text{o}}-{\left(\stackrel{-}{\text{G}\text{W}\text{L}}\right)}_{\text{i}\text{o}})}^{2}{\sum\:}_{\text{i}=1}^{\text{n}}{({\left(\text{G}\text{W}\text{L}\right)}_{\text{i}\text{p}}-{\left(\stackrel{-}{\text{G}\text{W}\text{L}}\right)}_{\text{i}\text{p}})}^{2}}}$$

3 $$\:\text{N}\text{S}\text{E}=1-\frac{{\sum\:}_{\text{i}=1}^{\text{n}}{\left(\right(\text{G}\text{W}\text{L}{)}_{\text{i}\text{o}}-\left(\text{G}\text{W}\text{L}{)}_{\text{i}\text{p}}\right)}^{2}}{{\sum\:}_{\text{i}=1}^{\text{n}}{\left(\right(\text{G}\text{W}\text{L}{)}_{\text{i}\text{o}}-\left(\stackrel{-}{\text{G}\text{W}\text{L}}{)}_{\text{i}\text{o}}\right)}^{2}}$$

4 $$\:\text{R}\text{E}=1-\frac{|\left(\text{G}\text{W}\text{L}\right){)}_{\text{i}\text{o}}-\left(\text{G}\text{W}\text{L}\right){)}_{\text{i}\text{p}}|}{\left(\text{G}\text{W}\text{L}\right){)}_{\text{i}\text{o}}}$$

5 $$\:\text{R}\text{R}\text{S}\text{E}=\sqrt{\frac{{\sum\:}_{\text{i}=1}^{\text{n}}{\left(\right(\text{G}\text{W}\text{L}{)}_{\text{i}\text{o}}-\left(\text{G}\text{W}\text{L}{)}_{\text{i}\text{p}}\right)}^{2}}{{\sum\:}_{\text{i}=1}^{\text{n}}{\left(\right(\text{G}\text{W}\text{L}{)}_{\text{i}\text{o}}-\left(\stackrel{-}{\text{G}\text{W}\text{L}}{)}_{\text{i}\text{o}}\right)}^{2}}}$$

Where (GWL)_io_ = measured GWL data, (GWL)_ip_ = predicted GWL data, ($\:\stackrel{-}{\text{G}\text{W}\text{L}}$)_io_ = average of measured GWL data, ($\:\stackrel{-}{\text{G}\text{W}\text{L}}$)_ip_ = average of predicted GWL data, and n = total number of data utilized.

## Case study

This research employed the different meteorological and groundwater indicators for predicting GWLs in Bongseong well. As explained in the previous description, the evaluation measures of employed machine learning (i.e., SGB, RF, GRNN, and GMDH) and deep learning (i.e., Deep ESN and LSTM) models for predicting GWLs are the essence of the underlying research project.

### Predicting GWLs based on scenario 01 in Bongseong well

In the addressed research, scenario 01 was proposed to predict GWLs in Bongseong well of Aewol-eup utilizing daily rainfall data from the Aewol (1) station and meteorological data of daily air temperature, relative humidity, and wind speed from the Witse Oreum station. Also, it included daily GWL data from wells located in Sanga1, Sanga2, Sanga3, Eom1, Jangcheon1, Hagwi1, and Hagwi3 located near the Bongseong well. Therefore, the current GWLs in the Bongseong well can be predicted utilizing meteorological and GWL data in seven different wells (scenario 01).

The topic for predicting GWLs in Bongseong well by employing various MLMs and DLMs utilizing scenario 01 is implemented in Table 5 based on five evaluation measures (i.e., RMSE, CC, NSE, RE, and RRSE) during the training procedure. The predictive evaluation of the RF1 model was better than that of competing MLMs and DLMs in clearly predicting GWLs. In addition, the LSTM1 model accomplished the worst performance compared to competing MLMs and DLMs for predicting GWLs in Bongseong well.

**Table 5:** Performance of MLMs and DLMs utilizing scenario 01.

Data	Models	Evaluation measure				
		RMSE (m)	CC	NSE	RE	RRSE
Training	SGB1	0.115	0.999	0.998	20.717	0.041
	RF1	0.038	1.000	1.000	5.420	0.013
	GRNN1	0.300	0.994	0.988	69.040	0.107
	GMDH1	0.243	0.996	0.992	49.043	0.087
	Deep ESN1	0.172	0.998	0.996	35.142	0.061
	LSTM1	0.602	0.977	0.954	99.053	0.215
Testing	SGB1	0.417	0.995	0.990	6.909	0.102
	RF1	0.115	1.000	0.999	2.224	0.028
	GRNN1	0.346	0.996	0.993	11.155	0.085
	GMDH1	0.499	0.992	0.985	15.623	0.122
	Deep ESN1	0.819	0.984	0.960	24.103	0.201
	LSTM1	1.504	0.933	0.864	45.270	0.369

Table 5 explains the outputs for predicting GWLs in Bongseong which utilize different MLMs and DLMs based on scenario 01 during the testing procedure. It can be judged from Table 5 that the RF1 model (RMSE = 0.115 m, CC = 1.000, NSE = 0.999, RE = 2.224, and RRSE = 0.028) supplemented more excellent performance than those of competing MLMs and DLMs for predicting GWLs. Also, the LSTM1 model (RMSE = 1.504 m, CC = 0.933, NSE = 0.864, RE = 45.270, and RRSE = 0.369) produced the worst prediction of GWLs compared to those of competing MLMs and DLM in Bongseong well.

**Fig. 5:**
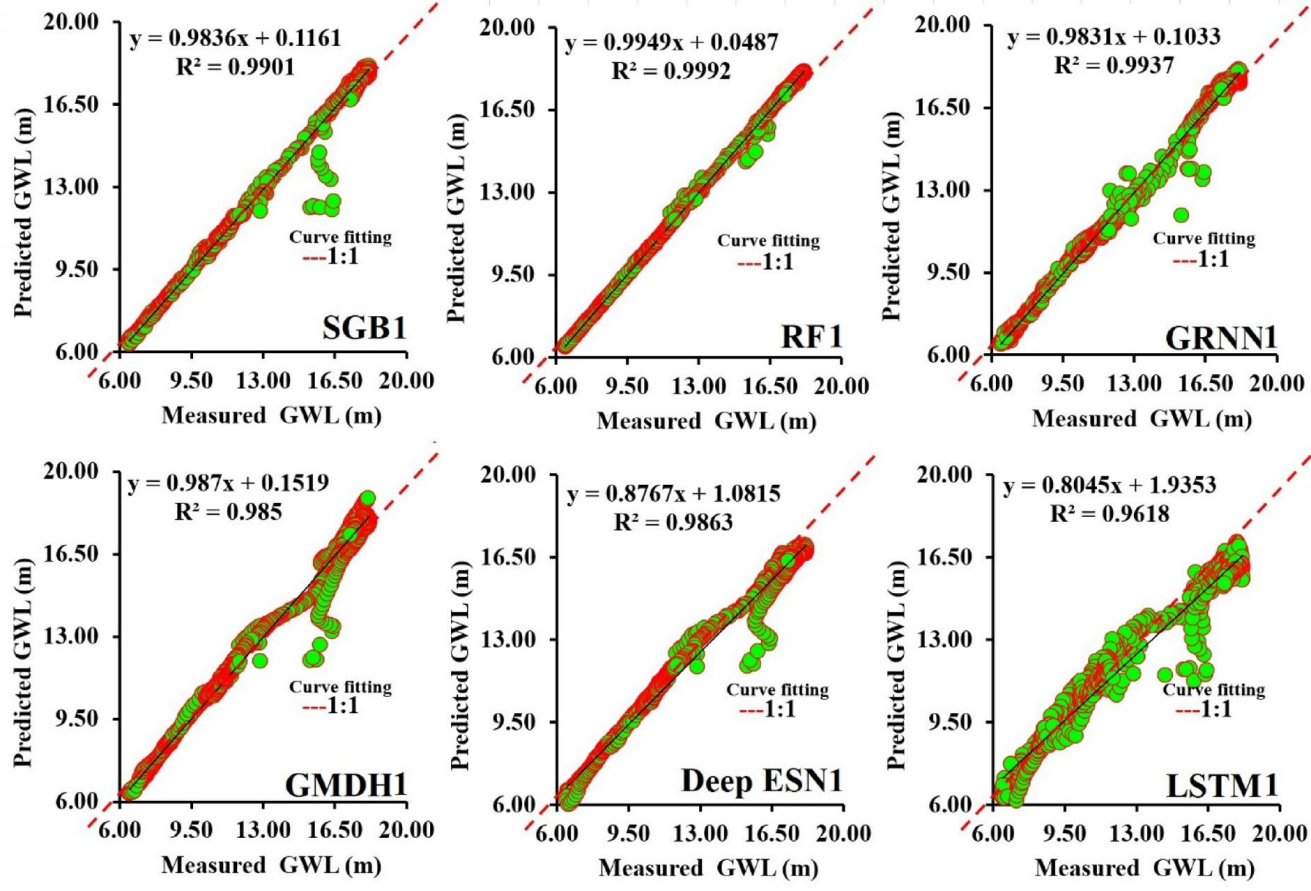
Comparison of performance between predicted and measured GWLs in scatterplots during testing procedure (scenario 01).

Figures 5a-f illustrate the scatterplots between predicted and measured GWL values utilizing SGB1, RF1, GRNN1, GMDH1, Deep ESN1, and LSTM1 models during the testing procedure. The corresponding scatterplot involves the best equation with determination coefficient and solid (fitted) line, respectively^61^. Considering each determinant coefficient, the RF1 model (R^2^ = 0.9992) suggested a higher value compared to competing SGB1, GRNN1, GMDH1, Deep ESN1, and LSTM1 models.

To display the predicted accuracy based on diverse visual assistance, boxplot^62^, violin plot^63^, spider plot^64^, and Taylor diagram^65^ were implemented to emphasize the predictive performance of employed MLMs and DLMs.

**Fig. 6:**
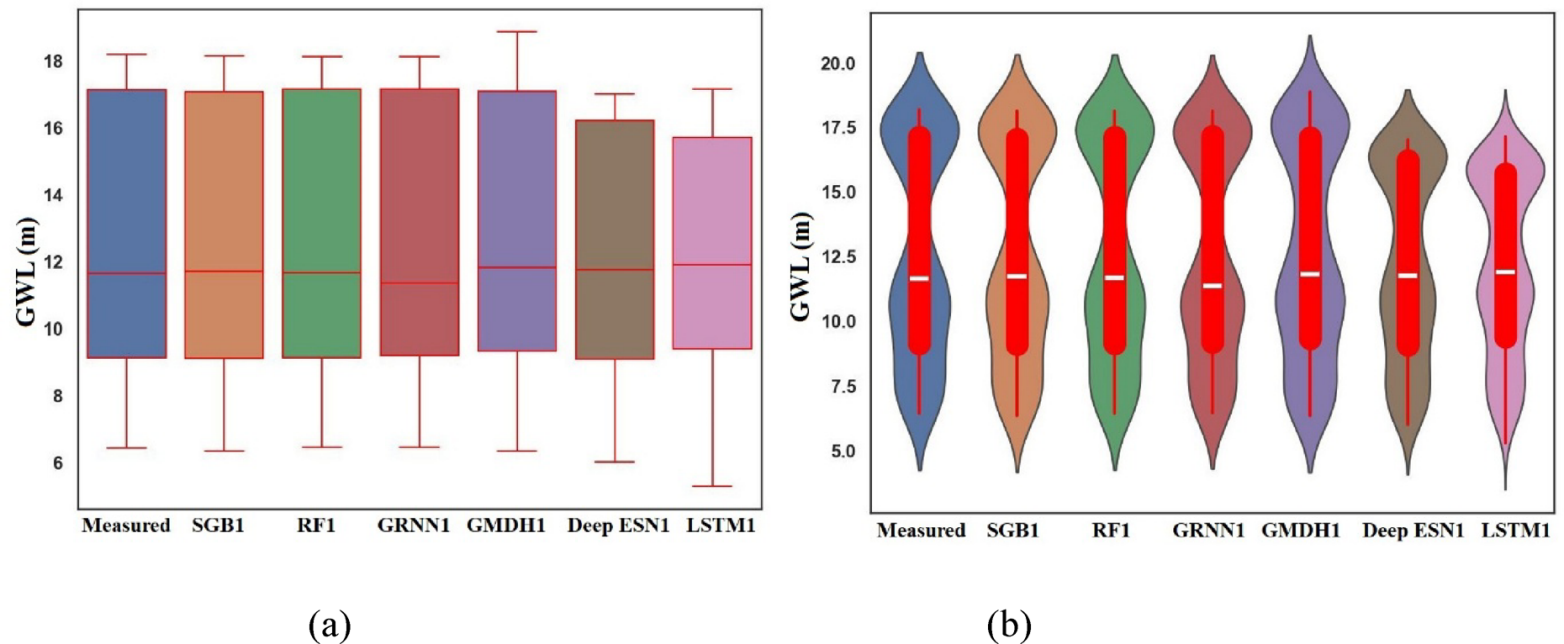
Boxplot and Violin plot between predicted and measured GWLs values utilizing different MLMs and DLMs during testing procedure (scenario 01). (**a**) Boxplot, (**b**) Violin plot.

Boxplot should be expressed as an optical approach for plotting the locality of predicted GWL values, spread, and skewness based on their quartiles^62,66^. Figure 6a provides the boxplots of measured, SGB1, RF1, GRNN1, GMDH1, Deep ESN1, and LSTM1 utilizing scenario 01 during the testing procedure. We determined from Fig. 6a that SGB1 and RF1 models can resemble the box shape and size of measured GWLs values compared to competing GRNN1, GMDH1, Deep ESN1, and LSTM1 models utilizing scenario 01 during the testing procedure. It was also found that the LSTM1 model did not follow the box shape and size of measured GWL values at all in Bongseong well.

A violin plot can be a visual assistance covering box diagrams based on displaying the arrangement of kernel density^63^. We verified from Fig. 6b that SGB1, RF1, and GRNN1 featured the shape pattern and size of measured GWLs values compared to competing GMDH1, Deep ESN1, and LSTM1 in Bongseong well during the testing procedure.

**Fig. 7:**
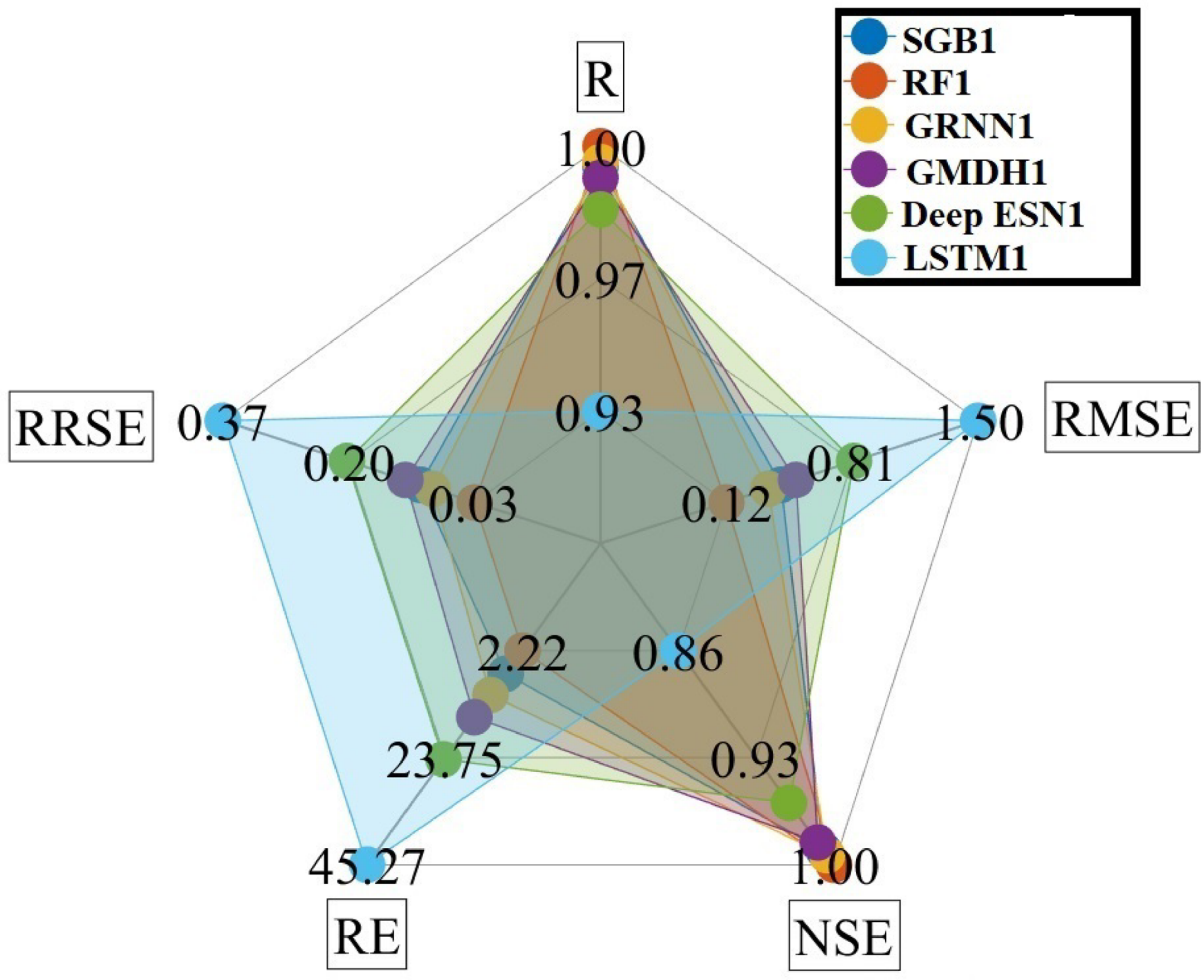
Spider plot between predicted and measured GWLs values utilizing different MLMs and DLMs during testing procedure (scenario 01).

Spider plot is one of the two-dimensional plotting methods for arranging values of evaluation measures^2,64^. The research addressed five evaluation measures utilizing RMSE, CC, NSE, RE, and RRSE diagrams. It can be judged from Fig. 7 that the RF1 model provided the most accurate prediction for five evaluation measures compared to competing MLMs and DLMs based on scenario 01 in Bongseong well during the testing procedure.

**Fig. 8:**
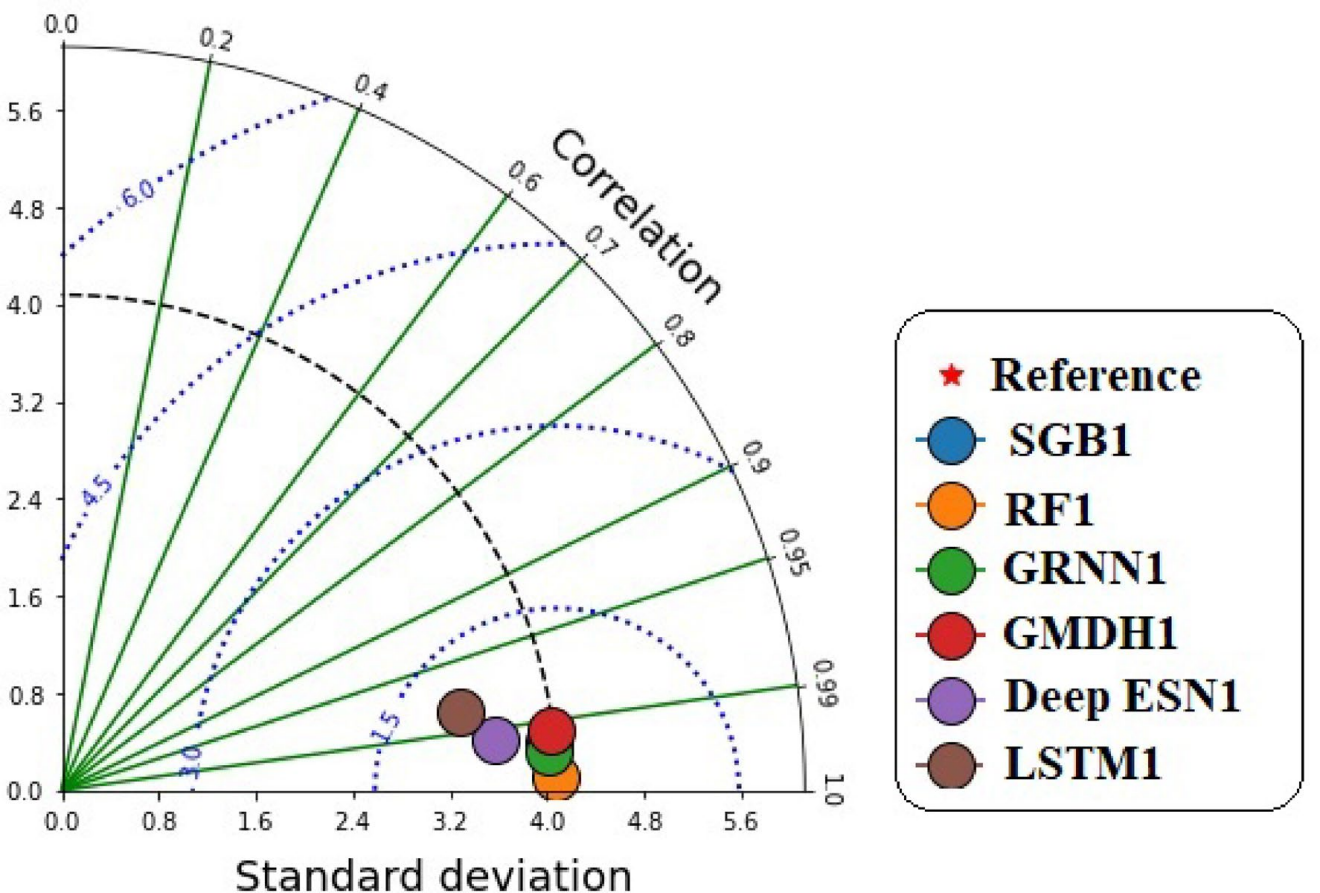
Taylor diagram between predicted and measured GWLs values utilizing different MLMs and DLMs during testing procedure (scenario 01).

Taylor diagram (Fig. 8) can display the values of predictive accuracy with the corresponding measured GWLs values utilizing correlation coefficient and standard deviation based on scenario 01. Figure 8 displayed that the point of the RF1 model gave the shortest distance to the reference point compared to competing MLMs and DLMs, while LSTM1 had the longest path from the reference point during the testing procedure.

### Predicting GWLs based on scenario 02 in Bongseong well

In the addressed research, scenario 02 was employed to predict GWLs in Bongseong well of Aewol-eup utilizing daily rainfall data from the Aewol (1) station and meteorological data of daily air temperature, relative humidity, and wind speed from the Witse Oreum station. Also, it adds up various groundwater indicators such as groundwater temperature, conductivity, and pressure measured from Bongseong well. Therefore, the current GWLs in Bongseong well can be predicted utilizing meteorological and groundwater indicators (scenario 02).

The issues for predicting GWLs in Bongseong well by applying different MLMs and DLMs utilizing scenario 02 are implemented in Table 6 based on five evaluation measures (i.e., RMSE, CC, NSE, RE, and RRSE) during the training procedure. The predictive evaluation of the GRNN2 model was better than that of competing MLMs and DLMs for predicting GWLs. Also, the LSTM2 model attained the worst accomplishment compared to competing MLMs and DLMs for predicting GWLs in Bongseong well.

**Table 6:** Performance of MLMs and DLMs utilizing scenario 02.

Data	Models	Evaluation measure				
		RMSE (m)	CC	NSE	RE	RRSE
Training	SGB2	1.507	0.843	0.709	301.553	0.539
	RF2	0.597	0.983	0.954	116.986	0.214
	GRNN2	0.278	0.995	0.990	30.185	0.099
	GMDH2	2.391	0.529	0.267	566.935	0.856
	Deep ESN2	2.342	0.545	0.297	513.788	0.838
	LSTM2	2.479	0.461	0.213	538.846	0.887
Testing	SGB2	1.898	0.885	0.783	70.088	0.466
	RF2	0.816	0.984	0.960	29.306	0.200
	GRNN2	0.443	0.951	0.996	9.585	0.065
	GMDH2	3.322	0.604	0.335	125.023	0.815
	Deep ESN2	5.900	0.011	0.247	199.496	0.868
	LSTM2	5.397	-0.249	0.370	215.252	0.794

In addition, Table 6 demonstrates the performance results for predicting GWLs in Bongseong, utilizing different MLMs and DLMs based on scenario 02 during the testing procedure. It can be concluded from Table 6 that the GRNN2 model (RMSE = 0.443 m, CC = 0.951, NSE = 0.996, RE = 9.585, and RRSE = 0.065) enhanced more outstanding performance than those of competing MLMs and DLMs for predicting GWLs clearly. In addition, the Deep ESN2 model (RMSE = 5.900 m, CC = 0.011, NSE = 0.247, RE = 199.496, and RRSE = 0.868) contributed the worst prediction of GWLs in Bongseong well compared to those of competing MLMs and DLM absolutely.

**Fig. 9:**
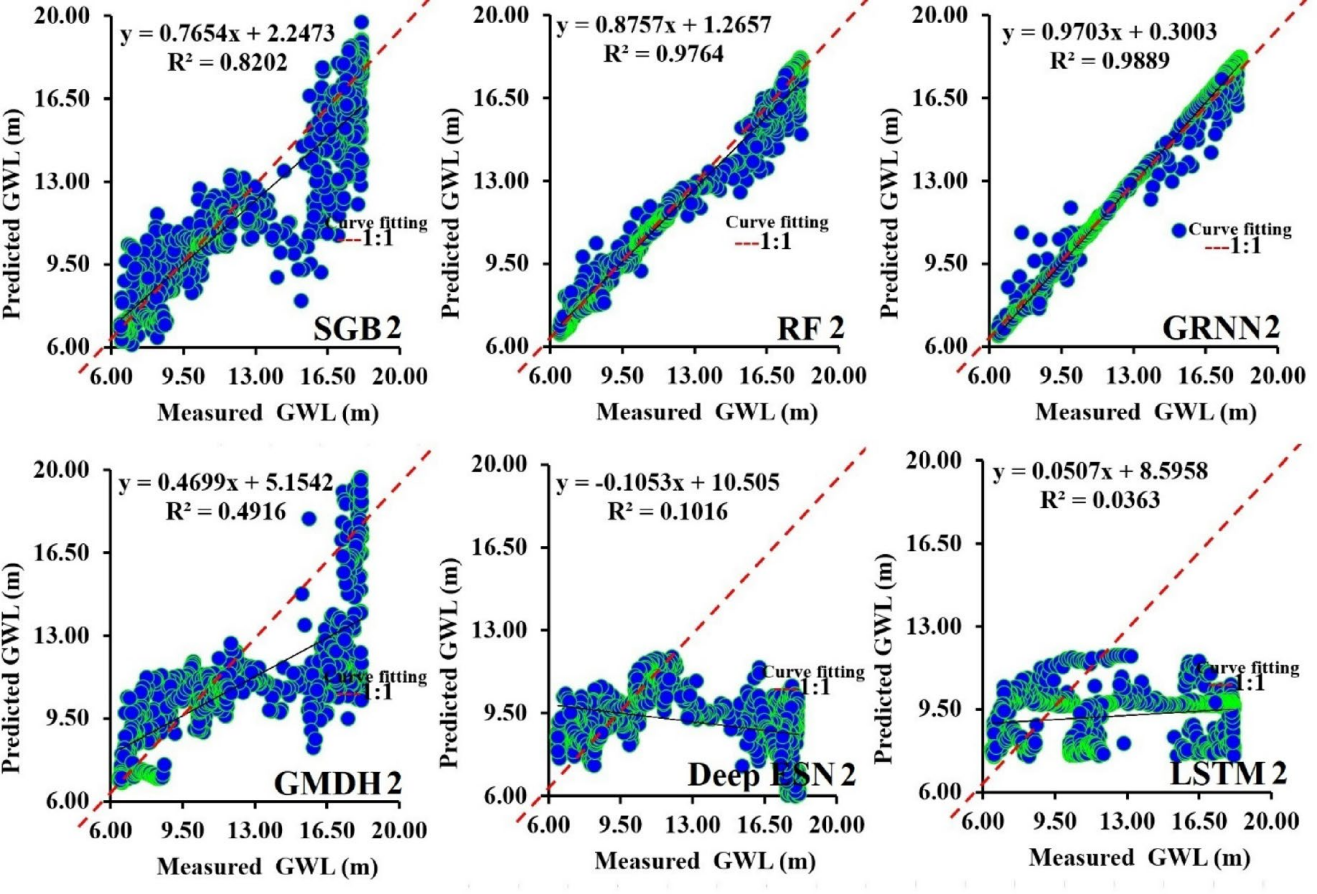
Comparison of performance between predicted and measured GWLs in scatterplots during testing procedure (scenario 02).

Figures 9a-f emphasize the scatterplot between predicted and measured GWL values utilizing SGB2, RF2, GRNN2, GMDH2, Deep ESN2, and LSTM2 models during the testing procedure. Recognizing each determinant coefficient, the GRNN2 model (R^2^ = 0.9889) provided higher accuracy compared to competing SGB2, RF2, GMDH2, Deep ESN2, and LSTM2 models. In the case of the Deep ESN2 model, it can be found that the slope for the linear function of the fitted line supplied a negative value (i.e., -0.1053).

**Fig. 10:**
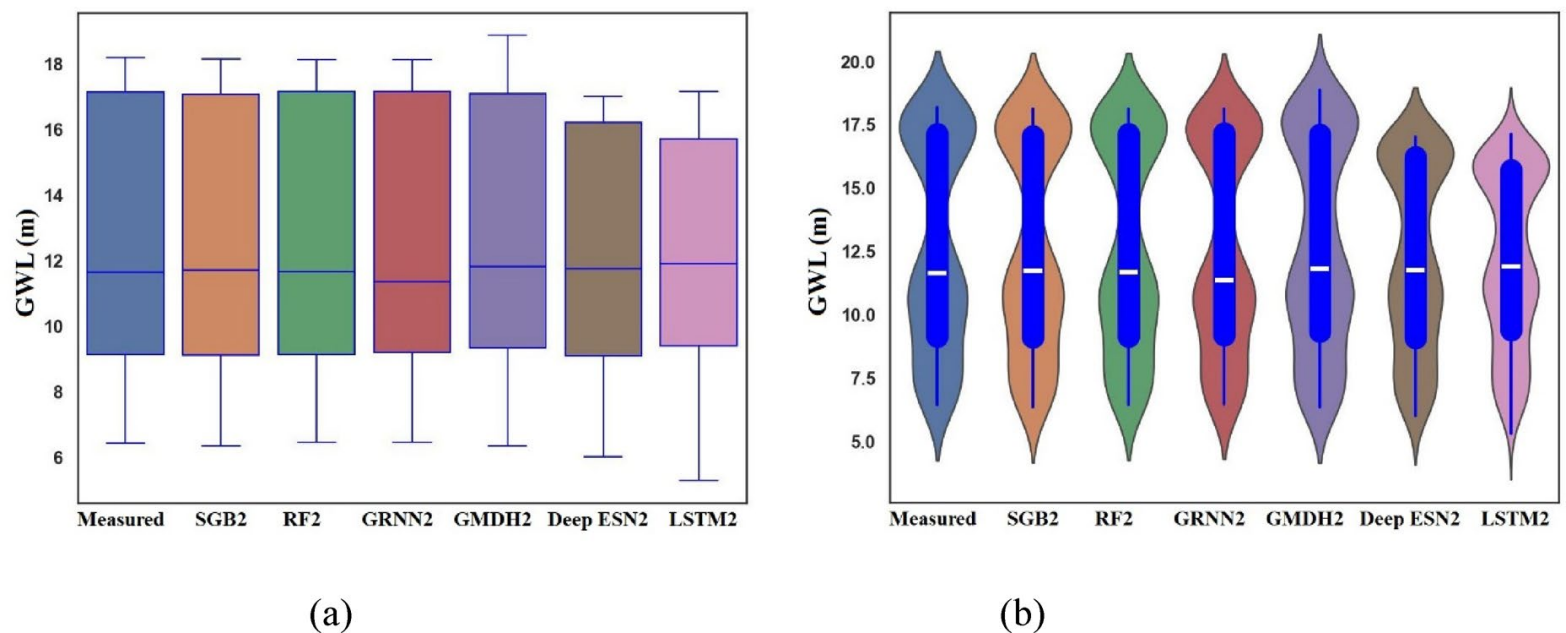
Boxplot and Violin plot between predicted and measured GWLs values utilizing different MLMs and DLMs during testing procedure (scenario 02). (**a**) Boxplot, (**b**) Violin plot.

Figure 10a furnishes the boxplots of measured, SGB2, RF2, GRNN2, GMDH2, Deep ESN2, and LSTM2 utilizing scenario 02 during the testing procedure. It can be chosen from Fig. 10a that SGB2 and RF2 models can resemble the box shape and size of measured GWLs values in Bongseong well definitely compared to competing GRNN2, GMDH2, Deep ESN2, and LSTM2 models utilizing scenario 02. It can also be judged that the LSTM2 model did not trace the box shape and size of measured GWL values in Bongseong well at all. In addition, the authors demonstrated from Fig. 10b that SGB2, RF2, and GRNN2 emphasized the shape pattern and size of measured GWL values in Bongseong well compared to competing GMDH2, Deep ESN2, and LSTM2 during the testing procedure.

**Fig. 11:**
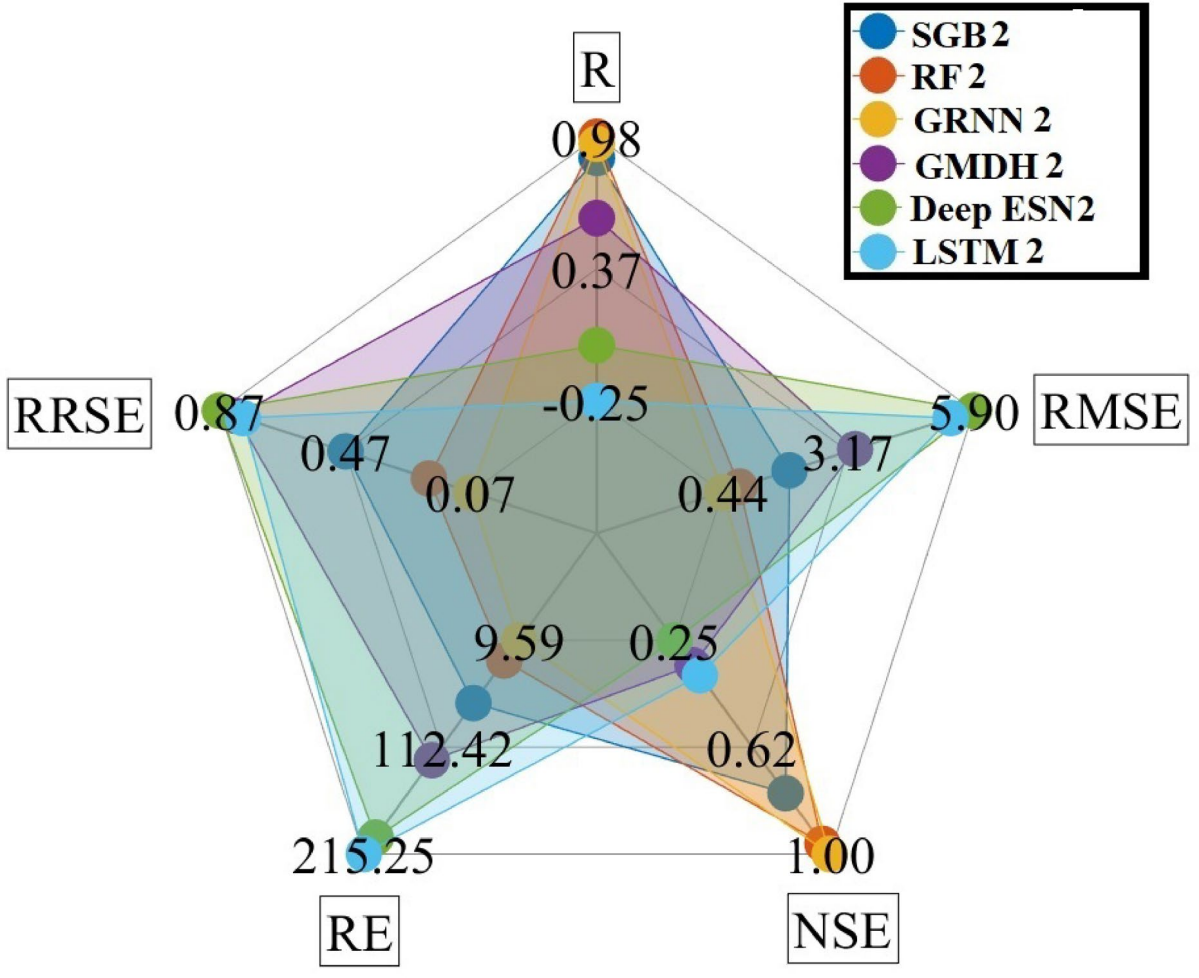
Spider plot between predicted and measured GWLs values utilizing different MLMs and DLMs during testing procedure (scenario 02).

Figure 11 explains the spider plot utilizing five evaluation measures, including RMSE, CC, NSE, RE, and RRSE diagrams, based on scenario 02. It can be inferred from Fig. 11 that the GRNN2 model provided the most accurate prediction for five evaluation measures compared to competing MLMs and DLMs during the testing procedure.

**Fig. 12:**
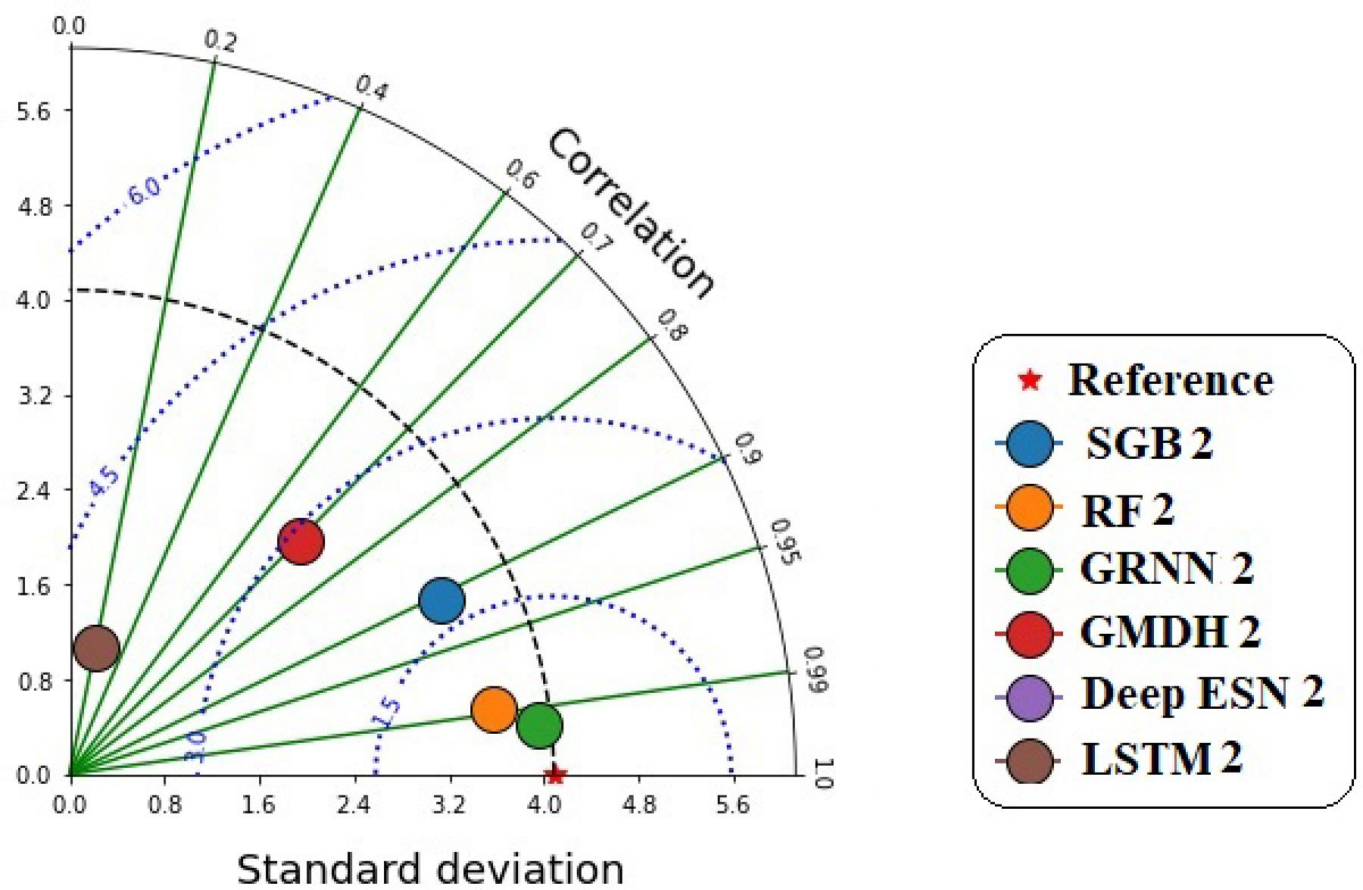
Taylor diagram between predicted and measured GWLs values utilizing different MLMs and DLMs during testing procedure (scenario 02).

Figure 12 illustrates the accurate values of prediction with the corresponding measured GWL values in Bongseong well utilizing correlation coefficient and standard deviation based on scenario 02 during the testing procedure. It provided that the point of GRNN2 model illustrated the shortest length to the reference point compared to competing MLMs and DLMs, while LSTM2 had the longest width from the reference point.

### Predicting GWLs based on scenario 03 in Bongseong well

In the addressed research, scenario 03 was implemented to predict GWLs in Bongseong well of Aewol-eup utilizing daily rainfall data from the Aewol (1) station and meteorological data of daily air temperature, relative humidity, and wind speed from the Witse Oreum station. Also, adding up the time series data of lead-time GWLs from 1-day (t-1) to 15-days (t-15) lead-time as input data of input layer, the current GWLs in Bongseong well were predicted utilizing meteorological and lead-time time series data of GWLs in Bongseong (scenario 03).

The issues for predicting GWLs in Bongseong well by handling different MLMs and DLMs utilizing scenario 03 are resolved in Table 7 based on five evaluation measures (i.e., RMSE, CC, NSE, RE, and RRSE) during the training procedure. The predictive evaluation of the RF3 model was superior to those of competing MLMs and DLMs for predicting GWLs perfectly. In addition, the GRNN3 model accomplished the worst achievement compared to those of competing MLMs and DLMs for predicting GWLs in Bongseong well.

**Table 7:** Performance of MLMs and DLMs utilizing scenario 03.

Data	Models	Evaluation criteria				
		RMSE (m)	CC	NSE	RE	RRSE
Training	SGB3	0.054	1.000	1.000	7.829	0.020
	RF3	0.024	1.000	1.000	3.392	0.008
	GRNN3	0.124	0.999	0.998	28.542	0.044
	GMDH3	0.049	1.000	1.000	7.600	0.018
	Deep ESN3	0.052	1.000	1.000	8.000	0.018
	LSTM3	0.034	1.000	1.000	7.058	0.012
Testing	SGB3	0.109	1.000	0.999	2.005	0.027
	RF3	0.053	1.000	1.000	1.114	0.013
	GRNN3	0.202	0.984	0.999	7.372	0.030
	GMDH3	0.097	1.000	0.999	1.703	0.024
	Deep ESN3	0.116	0.999	1.000	2.101	0.017
	LSTM3	0.251	0.999	0.999	6.443	0.037

Table 7 presents the statistical outcomes for predicting GWLs in Bongseong utilizing different MLMs and DLMs based on scenario 03 during the testing procedure. It can be confirmed from Table 7 that the RF3 model (RMSE = 0.053 m, CC = 1.000, NSE = 1.000, RE = 1.114, and RRSE = 0.013) boosted more distinguished performance than those of competing MLMs and DLMs for predicting GWLs certainly. In addition, the LSTM3 model (RMSE = 0.251 m, CC = 0.999, NSE = 0.999, RE = 6.443, and RRSE = 0.037) supplied the worst prediction of GWLs compared to competing MLMs and DLM in Bongseong well during the testing procedure.

**Fig. 13:**
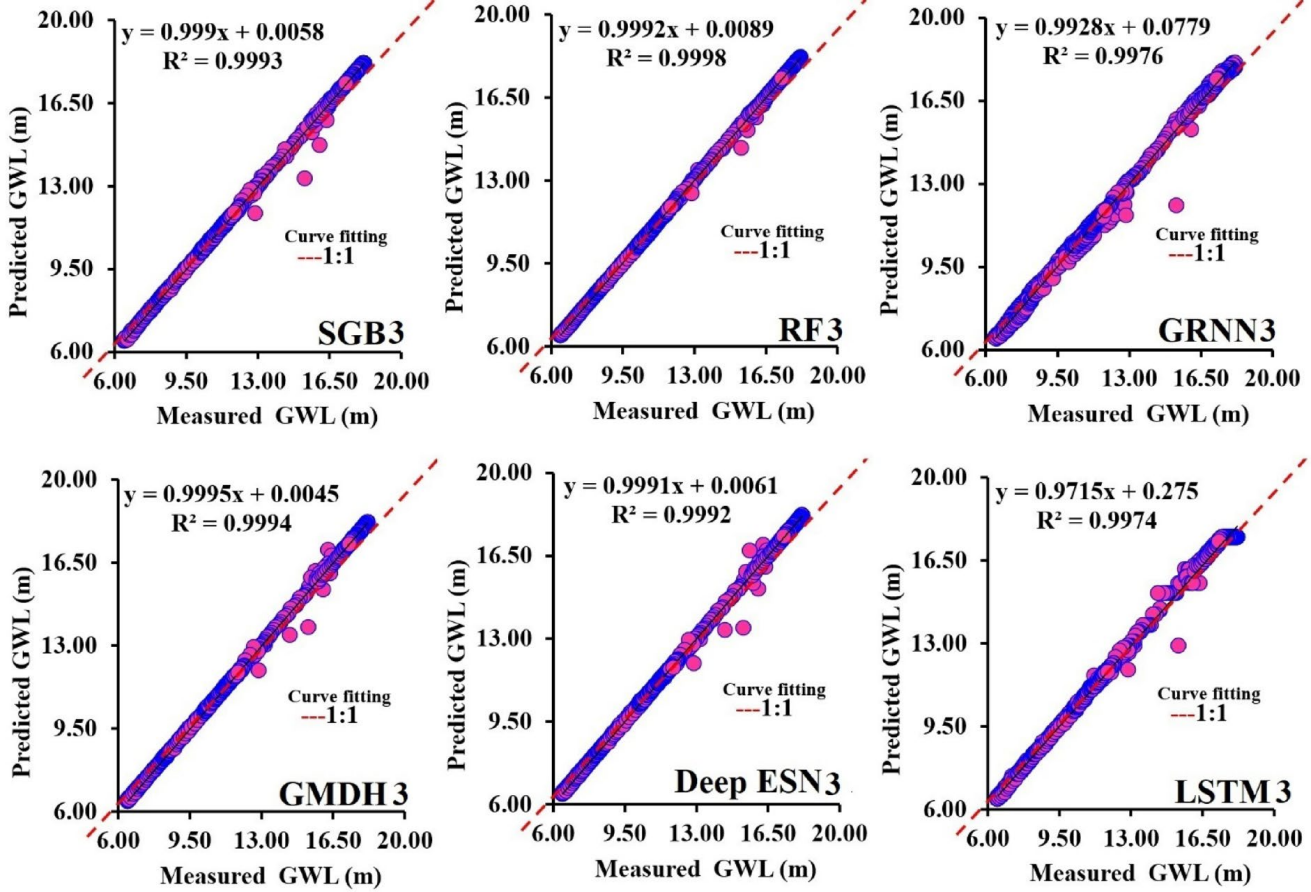
Comparison of performance between predicted and measured GWLs in scatterplots during testing procedure (scenario 03).

Figures 13a-f highlight the scatterplot between predicted and measured GWL values utilizing SGB3, RF3, GRNN3, GMDH3, Deep ESN3, and LSTM3 models during the testing procedure. Considering each determinant coefficient, the RF3 model (R^2^ = 0.9998) illustrated higher predictive accuracy compared to competing SGB3, GRNN3, GMDH3, Deep ESN3, and LSTM3 models.

**Fig. 14:**
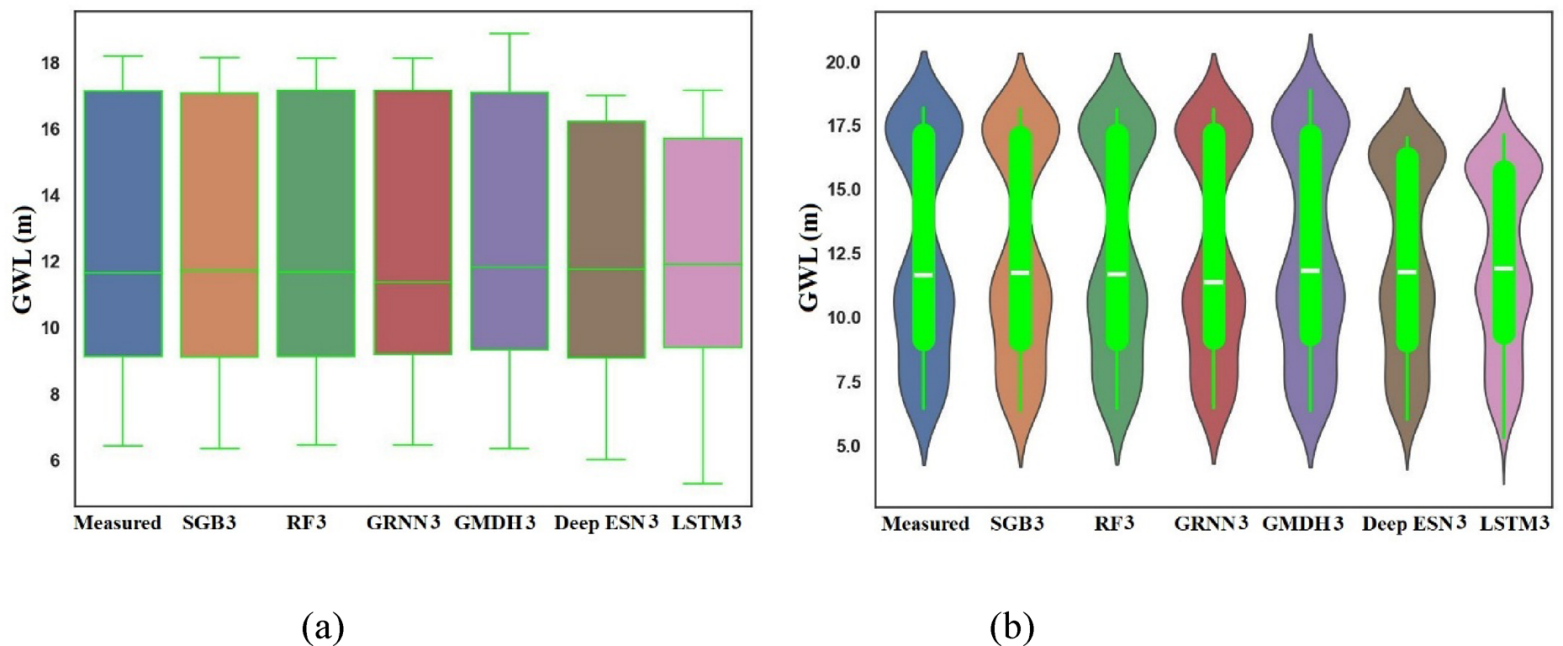
Boxplot and Violin plot between predicted and measured GWLs values utilizing different MLMs and DLMs during testing procedure (scenario 03). (**a**) Boxplot, (**b**) Violin plot.

Figure 14a decorates the boxplots of measured, SGB3, RF3, GRNN3, GMDH3, Deep ESN3, and LSTM3 utilizing scenario 03 during the testing procedure. It can be arranged from Fig. 14a that SGB3 and RF3 models can trace the box shape and size of measured GWLs values in Bongseong well surely compared to competing GRNN3, GMDH3, Deep ESN3, and LSTM3 models utilizing scenario 03. It also be assessed that LSTM3 model did not follow the box shape and size of measured GWL values in Bongseong well at all. In addition, it can be imagined from Fig. 14b that SGB3, RF3, and GRNN3 recommended the shape pattern and size of measured GWLs values in Bongseong well compared to competing GMDH3, Deep ESN3, and LSTM3.

**Fig. 15:**
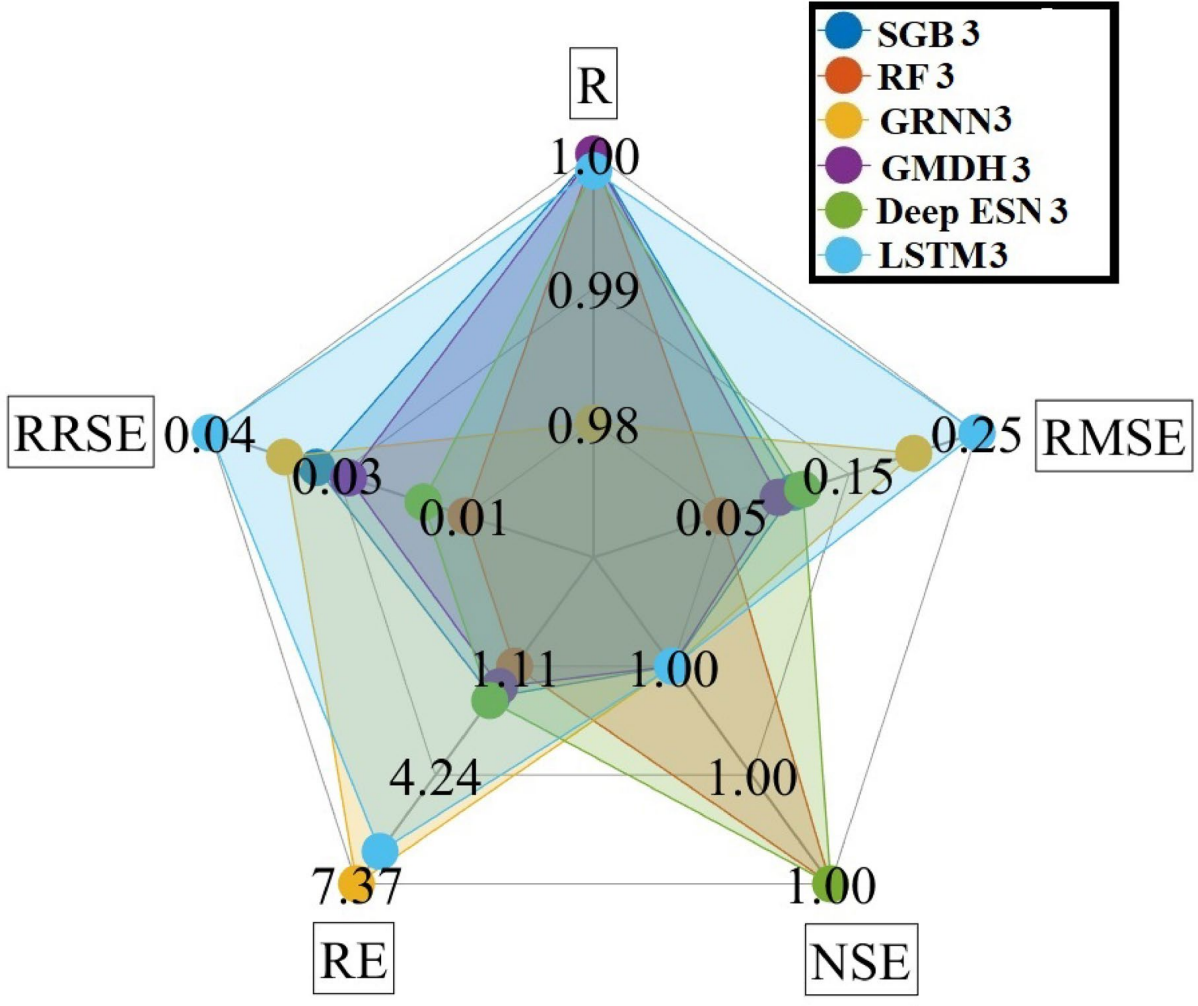
Spider plot between predicted and measured GWLs values utilizing different MLMs and DLMs during testing procedure (scenario 03).

Figure 15 provides the spider plot utilizing five evaluation measures, including RMSE, CC, NSE, RE, and RRSE diagrams, based on scenario 03. It can be suggested from Fig. 15 that the RF3 model gave the best accurate prediction for five evaluation measures compared to competing MLMs and DLMs during the testing procedure.

**Fig. 16:**
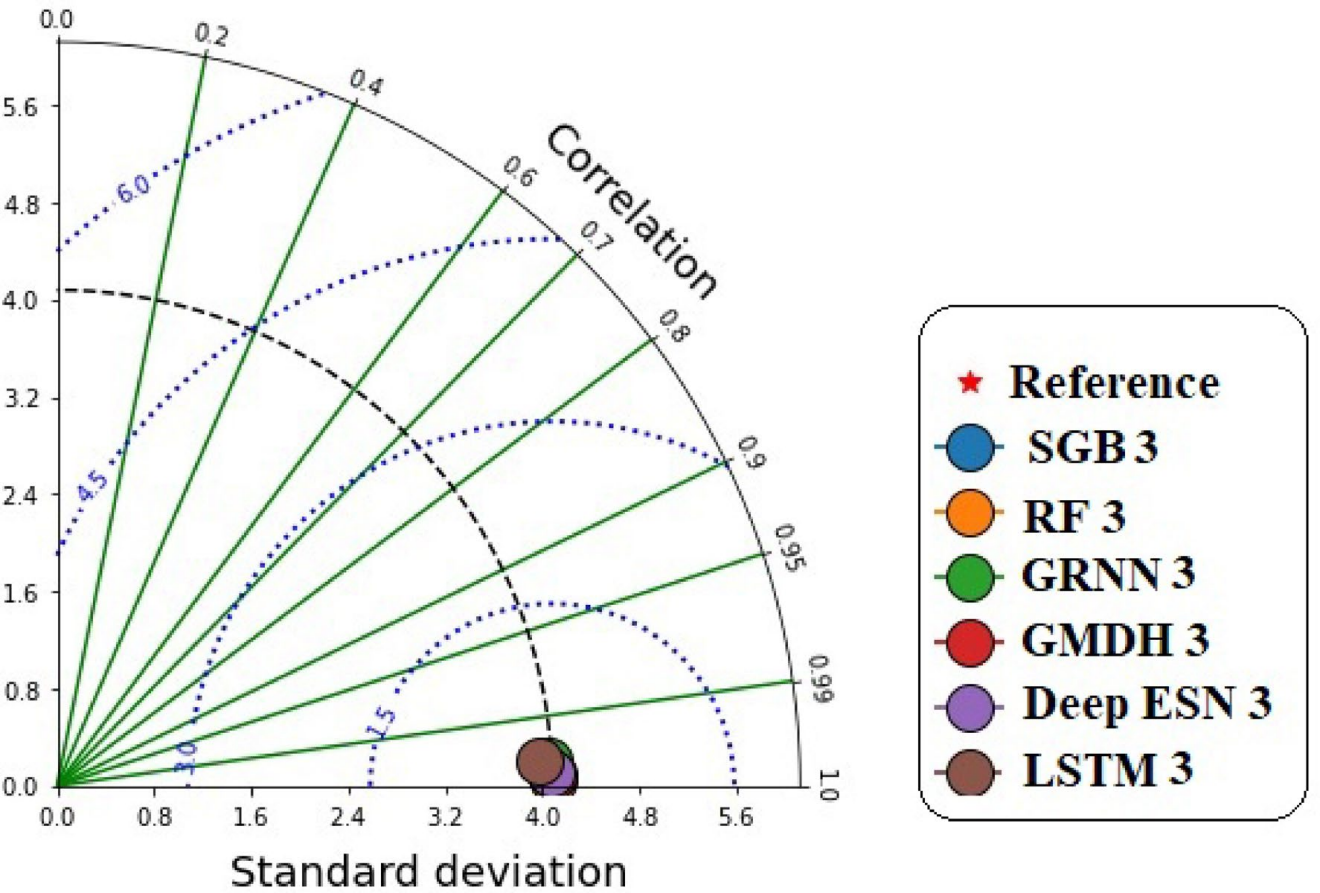
Taylor diagram between predicted and measured GWLs values utilizing different MLMs and DLMs during testing procedure (scenario 03).

Figure 16 discloses the accurate prediction values with the corresponding measured GWLs values in Bongseong well utilizing the correlation coefficient and standard deviation based on scenario 03. It provided that the points of all MLMs and DLMs demonstrated the shortest length to the reference point.

### Interpreting model prediction utilizing SHAP strategy

SHAP (SHapley Additive exPlanations) strategy can be described as a methodology to interpret the predictive accuracy of employed MLMs and DLMs based on the concept of sensitivity analysis. In MLMs and DLMs, each feature indicator is assigned a significant indicator explaining its contribution to the performance of employed MLMs and DLMs. It is a tremendous approach to obtain an increased comprehension of how feature data contribute to the selection of MLMs and DLMs^67^.

SHAP strategy provides the global and local interpretation. Global interpretation employs the importance of feature indicators and summary plots as a method of visual plotting. The importance of the feature indicator can be computed as the mean absolute SHAP value (i.e., average impact on model output magnitude). Accurate calculation for SHAP values requires all possible combinations of feature indicators, resulting in an exponentially large number of possible combinations for feature indications. That is, for a model with *n* feature indicator, calculating SHAP values for individual feature indicator requires calculating *2*^*n*^ possible feature subsets^68^. Also, an important plot of feature indicators organizes the most significant feature indicators in decreasing sequences. In addition, the leading feature indicator grants more predictive ability of MLMs and DLMs than the basic feature indicator.

In the case of summary plots, the x-axis characterizes the SHAP value (i.e., impact on model output), whereas the y-axis describes the feature value. Also, the color of the feature value can be changed from blue to red based on their importance. Red color implies a high effect, and blue color describes a low effect. That is, the addressed feature indicators in the summary plot are ranked likewise their predictive ability, and the graphs illustrate how changes in their values affect the predictive ability of MLMs and DLMs^69,70^.

**Fig. 17:**
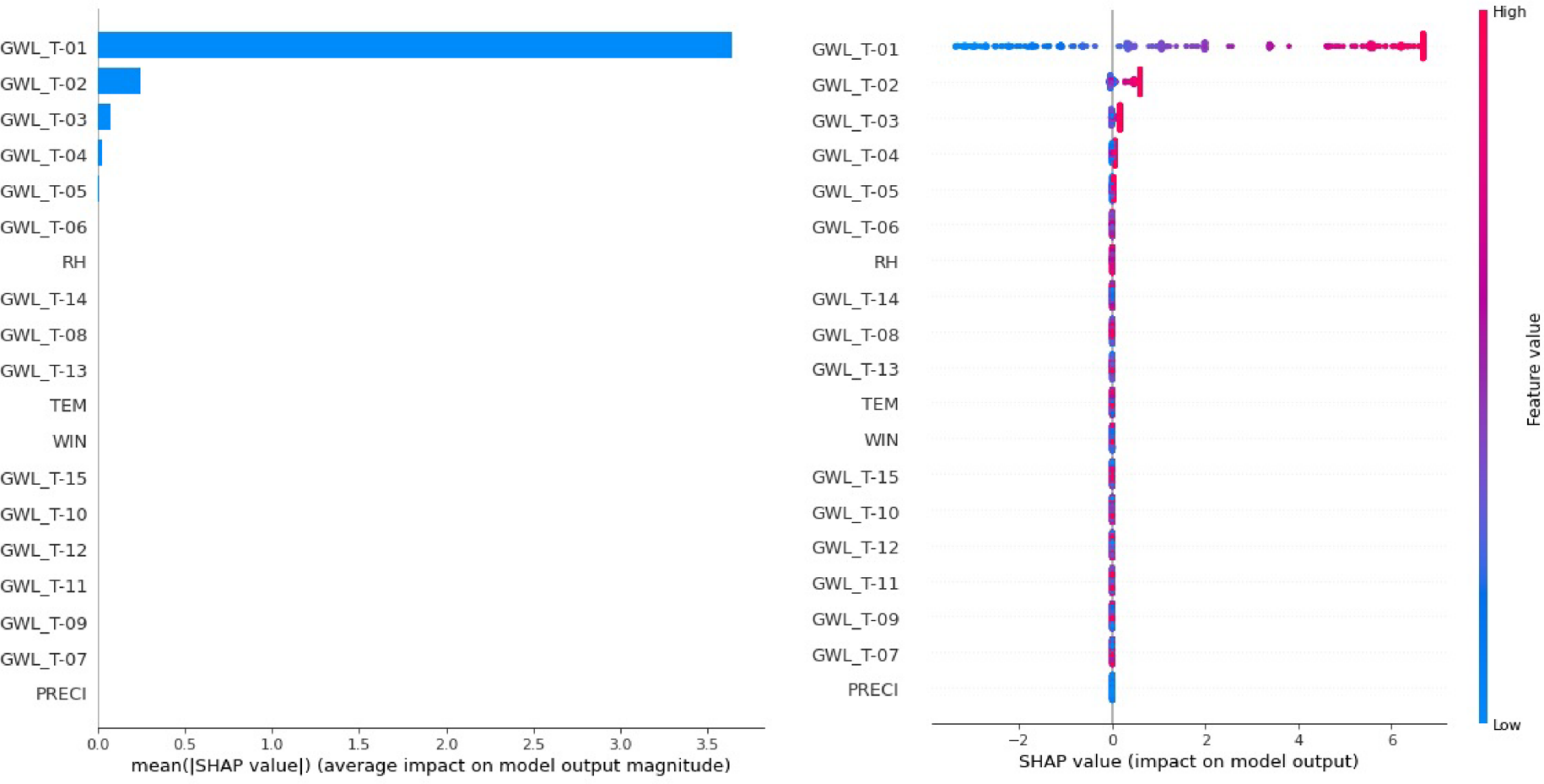
Global interpretation utilizing SHAP strategy based on feature importance (left panel) and summary plot (right panel) of the best model (RF3 model).

Figure 17 provided the global interpretation using the SHAP strategy based on feature importance and summary plot for the best model (i.e., RF3) among the employed MLMs and DLMs. From the importance plots of feature indicators (left panel), the feature indicator of GWL_T-01 (i.e., 1-day lead-time) provided the leading positive impact on the predictive ability of GWLs in Bongseong well for the RF3 model. This implies that the feature indicator of GWL_T-01 for the RF3 model could increase the predictive ability of GWLs obviously in Bongseong well during the testing procedure compared to the remaining feature indicators.

Depending on the summary plots (right panel) for the RF3 model, the feature indicators influencing GWL prediction can be ordered as GWL_T-01, GWL_T-02, GWL_T-03 and so on. The low value of SHAP (i.e., SHAP < 0) in the feature indicator of GWL_T-01 decreased the predictive ability of GWLs, whereas the large value of SHAP (i.e., SHAP > 4) in the feature indicator of GWL_T-01 increased the predictive ability of GWLs for RF3 model. The feature indicator of GWL_T-01 has a high and positive effect on the prediction of GWLs in Bongseong well for the RF3 model for SHAP ranges over 4.

**Fig. 18:**
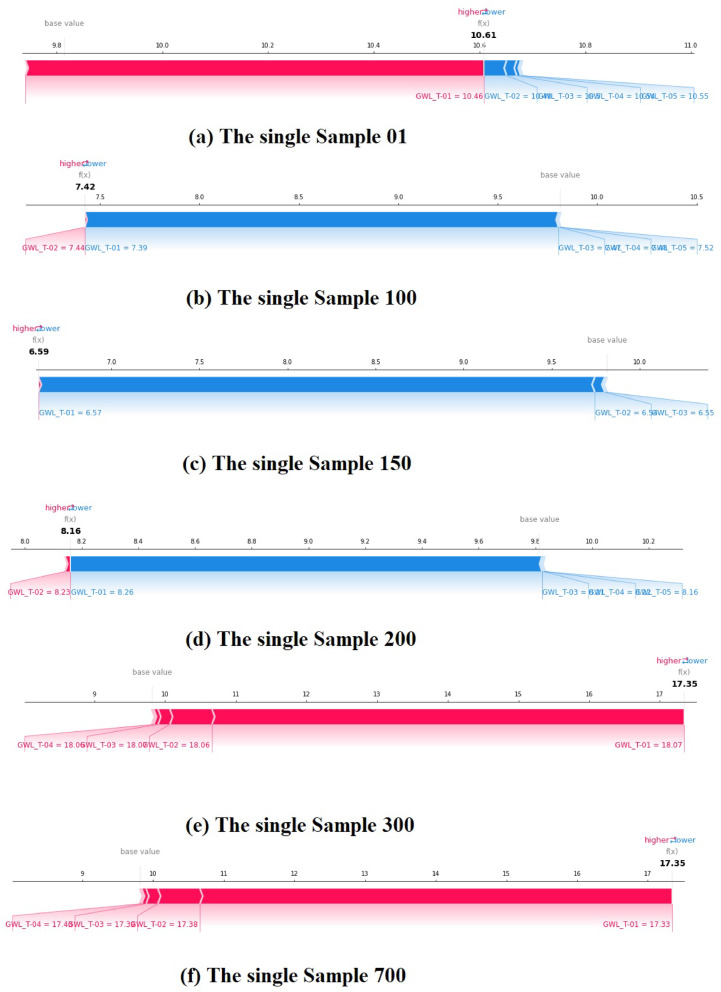
Local interpretation using SHAP strategy based on force plot for randomly selected single sample of the best model (RF3 model).

Local interpretation, however, implements force plots to illustrate corresponding SHAP for single MLM or DLM. Also, it tries to picture feature assignments at the sample grade to give more precise description and comprehension of a single MLM or DLM^71^. Figure 18 illustrates local interpretation using the SHAP strategy based on a force plot for the RF3 model (scenario 03). The force plot in Fig. 18 emphasizes the feature indicators for predicting GWLs in Bongseong well and forcing the predictive performance of the RF3 model from the value of the line to the actual value. The Red color indicates that a certain feature indicator presses the predictive accuracy higher, whereas the blue color indicates that an undergoing feature indicator presses the predictive accuracy lower. In all single samples (i.e., sample 01, 100, 150, 200, 300, and 700), the value of average model output (i.e., average predicted probability) provides 9.81 m.

In the force plot of single sample 01 for the RF3 model, the predicted value of GWLs in the Bongseong well is 10.61 m. The feature indicator of GWL_T-01 (10.46 m) could enhance the predictive ability of GWLs in Bongseong well, whereas the feature indicators of GWL_T-02 (10.48 m), GWL_T-03 (10.51 m), GWL_T-04 (10.54 m), and GWL_T-05 (10.55 m) could decrease the predictive ability of GWLs in Bongseong well.

Considering the force plot of single sample 100 for the RF3 model, the predicted value of GWLs in the Bongseong well is 7.42 m. The feature indicator of GWL_T-02 (7.44 m) could increase the predictive ability of GWLs in Bongseong well, whereas the feature indicators of GWL_T-01 (7.39 m), GWL_T-03 (7.42 m), GWL_T-04 (7.48 m), and GWL_T-05 (7.52 m) could reduce the predictive ability of GWLs in Bongseong well.

Recognizing the force plot of single sample 150 for the RF3 model, the predicted value of GWLs in the Bongseong well is 6.59 m. Any feature indicator could not press the predictive ability of GWLs in Bongseong well higher, whereas the feature indicators of GWL_T-01 (6.57 m), GWL_T-02 (6.56 m), and GWL_T-03 (6.55 m) pressed the predictive ability of GWLs in Bongseong well lower.

Depending on the force plot of single sample 200 for the RF3 model, the predicted value of GWLs in the Bongseong well is 8.16 m. The feature indicator of GWL_T-02 (8.23 m) stressed the predictive ability of GWLs in Bongseong well higher, whereas the feature indicators of GWL_T-01 (8.26 m), GWL_T-03 (8.21 m), GWL_T-04 (8.18 m), and GWL_T-05 (8.16 m) stressed the predictive ability of GWLs in Bongseong well lower.

Relying on the single samples 300 and 700 for the RF3 model, the predicted values of GWLs in the Bongseong well are 17.35 m in the same value. In addition, the feature indicators of GWL_T-01 (18.07 m (300) and 17.33 m (700)), GWL_T-02 (18.06 m (300) and 17.38 m (700)), GWL_T-03 (18.03 m (300) and 17.39 m (700)), and GWL_T-04 (18.02 m (300) and 17.42 m (700)) could increase the prediction of GWLs in Bongseong well. However, no feature indicators could decrease the predictive ability of GWLs in Bongseong well.

### One-way ANOVA test

One-way ANOVA (i.e., one-way analysis of variance) test presents a statistical method to compare the mean values of two or more samples whether they are significantly different based on computed F statistic and critical F statistic^72^. It can assess the significance of differences between the predicted and measured GWLs of MLMs and DLMs. Table 8 explains the results of the one-way ANOVA test of MLMs and DLMs utilizing all scenarios. In scenario 01, the predictive accuracy of MLMs was better than that of DLMs depending on the acceptance of the null hypothesis. The results of the one-way ANOVA test in scenario 02 provided the worst prediction of GWLs in Bongseong compared to scenarios 01 and 03 relying on the viewpoint of the null hypothesis. In addition, since all of the null hypothesis in scenario 03 demonstrated the acceptance of MLMs and DLMs, the developed models of scenario 03 were more powerful than those of scenarios 01 and 02. It can also be found from Table 8 that RF3 model provided the highest (computed) P value (0.993) with the lowest (computed) F statistic (6.9 × 10^− 5^) compared to the remaining MLMs and DLMs. Therefore, the RF3 model is the best model for predicting DWLs in Bongseong, as well as the competing MLMs and DLMs based on a one-way ANOVA test.

**Table 8:** Results for one-way ANOVA test of MLMs and DLMs utilizing all scenarios.

Division	Models	Level of Significance	One-way ANOVA test			Null Hypothesis (H_0_*)
			Computed P value	Computed F statistic	Critical F statistic	
Scenario 01	SGB1	0.05	0.669	0.183	3.848	Accept
	RF1	0.05	0.940	0.006	3.848	Accept
	GRNN1	0.05	0.604	0.270	3.848	Accept
	GMDH1	0.05	0.948	0.004	3.848	Accept
	Deep ESN1	0.05	0.017	5.720	3.848	Reject
	LSTM1	0.05	0.004	8.173	3.848	Reject
Scenario 02	SGB2	0.05	2.3 × 10^− 4^	13.632	3.848	Reject
	RF2	0.05	0.121	2.413	3.848	Accept
	GRNN2	0.05	0.717	0.132	3.848	Accept
	GMDH2	0.05	1.3 × 10^− 17^	74.950	3.848	Reject
	Deep ESN2	0.05	2.7 × 10^− 93^	489.876	3.848	Reject
	LSTM2	0.05	2.8 × 10^− 92^	483.661	3.848	Reject
Scenario 03	SGB3	0.05	0.976	9.2 × 10^− 4^	3.848	Accept
	RF3	0.05	0.993	6.9 × 10^− 5^	3.848	Accept
	GRNN3	0.05	0.948	0.004	3.848	Accept
	GMDH3	0.05	0.991	1.2 × 10^− 4^	3.848	Accept
	Deep ESN3	0.05	0.979	6.8 × 10^− 4^	3.848	Accept
	LSTM3	0.05	0.679	0.172	3.848	Accept

H0* = there are same between predicted and measured mean values in GWLs.

## Discussion

The current article accomplished the predictive accuracy of GWLs in Bongseong by employing different MLMs and DLMs based on the evaluation measures and various visual assistance. In the addressed research, three scenarios were employed to predict and forecast GWLs in the Bongseong well of Aewol-eup, Jeju Island. In addition, data on daily time scale for all scenarios (i.e., scenarios 01, 02, and 03) were determined from June 1, 2011, to December 31, 2020.

The first scenario (scenario 01) was applied to predict GWLs in Bongseong well utilizing meteorological data (i.e., rainfall, air temperature, relative humidity, and wind speed) and GWLs data of 7 different wells (i.e., in Sanga1, Sanga2, Sanga3, Eom1, Jangcheon1, Hagwi1, and Hagwi3). The second scenario (scenario 02) was implemented to predict GWLs in Bongseong well utilizing meteorological data (i.e., rainfall, air temperature, relative humidity, and wind speed) and groundwater indicators (i.e., temperature, conductivity, and pressure) in Bongseong well. Finally, the third scenario (scenario 03) was employed to predict GWLs in Bongseong utilizing meteorological data (i.e., rainfall, air temperature, relative humidity, and wind speed) and GWLs time series data with lead-time (i.e., from t-1 to t-15) in Bongseong. This structure allowed for an evaluation of both spatial and temporal influence factors on GWL predictions, enabling a comparative insight into different data representation schemes.

In scenario 01, the predictive results of training and testing procedures illustrated that the RF1 model was the best, and the LSTM1 model had the lowest predictive accuracy. In addition, scenario 02 demonstrated that the predictive accuracy of the GRNN2 model was the best, and the LSTM2 model (training) and Deep ESN2 model (testing) had the lowest predictive accuracy in training and testing procedures. Finally, in scenario 03, the RF3 model provided the best accuracy for predicting GWLs based on training and testing performances, whereas the GRNN3 model (training) and LSTM3 model (testing) were analyzed to be the lowest for predicting GWLs in Bongseong well. The clear decline in DL model performance under Scenario 02 may reflect their higher sensitivity to feature richness and temporal depth, highlighting the need for carefully engineered input data when applying these methods to groundwater problems.

Judging by each scenario, the predictive accuracy of MLMs and DLMs in scenario 03 led to better predictive accuracy than those of scenarios 01 and 02 concerning training and test data. In addition, the predictive accuracy for scenarios 01 and 03, which included GWLs time series for monitoring wells as input data, was superior to the predictive accuracy for scenario 02, which did not involve GWLs time series for monitoring wells. These results confirm that temporal memory within the groundwater system—captured through lagged GWLs—plays a dominant role in accurate forecasting. This observation aligns with prior findings in time-series-based GWL modeling studies^9,23^.

In particular, the predictive accuracy of scenario 03, including GWLs time series data with lead-time in Bongseong well, was evaluated to be superior to that of scenario 01, which involved GWLs time series data with seven different wells. In addition, as a result of accomplishing a sensitivity analysis on the best model (i.e., RF3) utilizing the SHAP strategy, it was found that the input indicator that most influenced the predictive accuracy of the RF3 model was demonstrated as 1-day lead-time GWLs (GWL_T-01) data in Bongseong well. This also confirms the effectiveness of SHAP for identifying key features and quantifying their contribution, addressing the interpretability gap that is common in black-box AI models. Feature importance plots and force plots offer actionable insights into the behavior of the model and can assist stakeholders in understanding prediction drivers.

Contributing the optimal MLMs and DLMs based on individual RMSE values during the testing procedure, RF3, which performed the best accurate prediction among all scenario, increased the predictive accuracy of GWLs in Bongseong well by 116.98% (RF1), 552.83% (GRNN1), 1,439.62% (RF2), 737.74% (GRNN2), and 83.02% (GMDH3), respectively. Such substantial performance improvement underscores the suitability of ensemble models like RF when sufficient high-quality temporal data is available, especially in regions with hydrological variability like Jeju Island.

Considering previous literature similar to the addressed research, Sahoo et al.^73^ tried to model GWLs employing spectral analysis, a machine learning approach, and uncertainty analysis at two locations, in the United States. Results showed that they provided a reliable analysis for GWL modeling utilizing a multilayer perceptron (MLP) approach. Afan et al. (2021) applied deep learning (DL) and ensemble deep learning (EDL) models to predict GWLs in five wells, in Malaysia. They employed two methods to predict GWLs. The first method employed four wells as input indicators, and one well was selected as an output indicator. For the second method, time series data on the 20-day lead time of five wells were selected as input indicators. As a result, they provided that the first method demonstrated that the EDL model had a better prediction of GWLs than DLM, except for one well (i.e., Paya Indah Wetland). Also, the second method illustrated that EDL model predicted GWLs better than DLM in all wells. Pham et al.^74^ developed six MLMs, including bagging-random tree (B-RT), bagging-random forest (B-RF), decision stump (DS), M5P, SVM, locally weighted linear regression (LWLR), and reduce error pruning tree (REPT), to predict GWLs, Bangladesh. Results explained that B-RT and B-RF models were selected best for predicting GWLs. Therefore, in the addressed research, the RF model employed for predicting GWLs time series was demonstrated as a superior model, leading to similar predictive results compared to the previous research. These comparative studies reinforce the reliability of ensemble learning frameworks such as RF in groundwater studies, further validating our findings within the broader context of data-driven hydrological modeling.

Some scientists demand an appropriate scale (e.g., global or local) to assess groundwater issues including resources, operation, and management and so on. One reason, why a global groundwater perspective is essentially required, is that it highlights the role of sustainability and improves our understanding for it^75^. However, different arguments for global groundwater sustainability are always dependent on regional and local hydrology field (e.g., hydrology for Jeju island). To solve the challenging issue for global groundwater sustainability, a lot of data on various problems, including groundwater resources, groundwater quality, custom, culture, politics, and laws in local boundaries (e.g., Jeju island), must be collected, and an assessment of global groundwater sustainability must be planned and evaluated based on diverse datasets.

## Conclusion

In the addressed research, three scenarios are applied to predict GWLs in the Bongseong well of Aewol-eup, located on Jeju Island. The data on daily time scale for three scenarios (i.e., 01, 02, and 03) is utilized, and performance results of training and testing procedures are accomplished utilizing five evaluation measures.

Table 9 describes comparison of models ranking for three scenarios (i.e., 01, 02, and 03) based on the statistical results of five evaluation measures. For the first scenario, by providing statistical performance for the training and testing procedures, the RF1 model is evaluated as the outstanding model for predicting GWLs in Bongseong well. In the second scenario, by suggesting statistical achievement for the training and testing procedures, the GRNN2 model is selected as the superior model for predicting GWLs in Bongseong well. Finally, in the third scenario, by proposing statistical accomplishment for the training (RMSE = 0.024 m, CC = 1.000, NSE = 1.000, RE = 3.392, and RRSE = 0.008) and testing (RMSE = 0.053 m, CC = 1.000, NSE = 1.000, RE = 1.114, and RRSE = 0.013) procedures, the RF3 model is determined as the outstanding model for predicting GWLs in Bongseong well. Among the three scenarios and different MLMs and DLMs, the RF3 model in the third scenario is the best model for predicting GWLs in Bongseong well. In particular, for scenarios 01 and 03 that include GWL time series as feature indicators, RF models (i.e., RF1 and RF3) provide better predictive accuracy for GWLs in Bongseong well compared to remaining MLMs and DLMs.

**Table 9:** Comparison of models ranking for three scenarios.

Division	Data	Models Ranking
Scenario 01	Training	RF1 > SGB1 > Deep ESN1 > GMDH1 > GRNN1 > LSTM1
	Testing	RF1 > GRNN1 > SGB1 > GMDH1 > Deep ESN1 > LSTM1
Scenario 02	Training	GRNN2 > RF2 > SGB2 > Deep ESN2 > GMDH2 > LSTM2
	Testing	GRNN2 > RF2 > SGB2 > GMDH2 > Deep ESN2 > LSTM2
Scenario 03	Training	RF3 > LSTM3 > GMDH3 > Deep ESN3 > SGB3 > GRNN3
	Testing	RF3 > GMDH3 > SGB3 > Deep ESN3 > GRNN3 > LSTM3

As a result of accomplishing a sensitivity analysis using the SHAP strategy for the best model, RF3, it is found that the GWLs data of GWL_T-01 (1-day lead-time) clearly influences predictive accuracy. In order to provide more reliability for the predictive results of addressed research, it is judged that continued research utilizing various GWL data, MLMs, and DLMs is required. In addition, the one-way ANOVA test demonstrates that the RF3 model is the most robust with the highest P value and the lowest F statistic compared to comparable MLMs and DLMs.

The weakness and disadvantage of this research can be explained by the development of a few neuro-inspired models, including MLMs and DLMs, for predicting GWLs by utilizing restricted data samples. Since the accurate prediction issue of GWLs in Bongseong well has emphasized some MLMs (i.e., SGB, RF, GRNN, and GMDH) and DLMs (i.e., Deep ESN and LSTM), the current research cannot validate and confirm the predictive accuracy within the universal category. In addition, the geographic limitations of a single well location may limit the generalizability of results of this research, and therefore, further researches in diverse hydrogeological environments are needed.

Therefore, it is necessary to ensure the robustness of GWL prediction by performing diverse research utilizing more data collected and various MLMs and DLMs. In addition, the lack of different experiments can be improved by diverse researches, which integrates different neuro-inspired models, metaheuristic evolutionary algorithms, and data preprocessing methods, to confirm the best predictive accuracy of GWLs with high levels.

Although it is not a field of same research for predicting GWLs based on the state-of-the-art articles, the authors could find that, for example, Poursaeid^76^ developed ensemble machine learning (EML) combined with diverse meta-heuristic algorithms for predicting streamflow, USA. Also, Zhang et al.^77^ integrated graph convolutional networks (GCN) with an integrated hydrological and hydrodynamic model (DHHDM) for forecasting water level, China. Future attempts may also benefit from exploring transfer learning and domain adaptation to reutilize trained models across similar aquifer systems, thereby reducing data dependency while preserving accuracy.

## Data Availability

The data presented in this research are available upon request from the corresponding author (contact [ozgur.kisi@th-luebeck.de](mailto: ozgur.kisi@th-luebeck.de) ).
